# Orthosteric ligand selectivity and allosteric probe dependence at Hydroxycarboxylic acid receptor HCAR2

**DOI:** 10.1038/s41392-023-01625-y

**Published:** 2023-09-25

**Authors:** Lin Cheng, Suyue Sun, Heli Wang, Chang Zhao, Xiaowen Tian, Ying Liu, Ping Fu, Zhenhua Shao, Renjie Chai, Wei Yan

**Affiliations:** 1 Department of Otolaryngology Head and Neck Surgery, Sichuan Provincial People’s Hospital, University of Electronic Science and Technology of China, Chengdu, 610000 China; 2 Division of Nephrology and Kidney Research Institute, State Key Laboratory of Biotherapy, West China Hospital, Sichuan University, Chengdu, Sichuan 610041 China; 3 Frontiers Medical Center, Tianfu Jincheng Laboratory, Chengdu, 610212 China; 4 State Key Laboratory of Digital Medical Engineering, Department of Otolaryngology Head and Neck Surgery, Zhongda Hospital, School of Life Sciences and Technology, Advanced Institute for Life and Health, Jiangsu Province High-Tech Key Laboratory for Bio-Medical Research, Southeast University, Nanjing, 210096 China; 5 Co-Innovation Center of Neuroregeneration, Nantong University, Nantong, 226001 China

**Keywords:** Structural biology, Target validation

## Abstract

Hydroxycarboxylic acid receptor 2 (HCAR2), a member of Class A G-protein-coupled receptor (GPCR) family, plays a pivotal role in anti-lipolytic and anti-inflammatory effects, establishing it as a significant therapeutic target for treating dyslipidemia and inflammatory diseases. However, the mechanism underlying the signaling of HCAR2 induced by various types of ligands remains elusive. In this study, we elucidate the cryo-electron microscopy (cryo-EM) structure of G_i_-coupled HCAR2 in complex with a selective agonist, MK-6892, resolved to a resolution of 2.60 Å. Our structural analysis reveals that MK-6892 occupies not only the orthosteric binding pocket (OBP) but also an extended binding pocket (EBP) within HCAR2. Pharmacological assays conducted in this study demonstrate that the OBP is a critical determinant for ligand selectivity among the HCARs subfamily. Moreover, we investigate the pharmacological properties of the allosteric modulator compound 9n, revealing its probe-dependent behavior on HCAR2 in response to varying orthosteric agonists. Collectively, our findings provide invaluable structural insights that contribute to a deeper understanding of the regulatory mechanisms governing HCAR2 signaling transduction mediated by both orthosteric and allosteric ligands.

## Introduction

GPCRs, the largest membrane protein superfamily on the cell surface, mediate distinct cellular signaling pathways and are associated with physiological processes of life, including vision, hearing, smell, feel and taste, as well as the development, maturation, and functioning for cells, tissues, organs.^1^ The important roles make GPCRs an attractive drug target for diseases treatment.^2^ The hydroxy-carboxylic acid (HCA) receptor family, belonging to Class A GPCR family, comprises three subtypes HCAR1, HCAR2, and HCAR3 that senses to metabolites.^3^ HCAR2 is widely expressed in diverse cell types, including adipocytes cells (white or brown adipocytes), immune cells (macrophages, dendritic cells) and so on.^4–6^ Previous literatures indicated that HCAR2 could be activated by the endogenous ligands: the ketone body β-hydroxybutyrate (β-HOB) and butyrate.^5,6^ In adipocytes cells, HCAR2 mediates the anti-lipolytic effect, which can decrease the level of plasma-free fatty acids (FFAs).^7^ The reduction of FFAs would subsequently slowdown the synthesis of total cholesterol, triglycerides, and low-density lipoprotein cholesterol (LDL-cholesterol), while simultaneously increase the high-density lipoprotein cholesterol (HDL-cholesterol) levels in the liver.^7^ In addition, recent studies suggest that HCAR2 activation results in beneficial anti-inflammatory effects in a range of diseases, including intestinal inflammation, colon cancer, and neurologic diseases.^6,8^ In LPS-induced monocytes or macrophages, we demonstrated that the activation of HCAR2 suppresses the expression levels of several pro-inflammatory cytokines, including tumor necrosis factor alpha (TNF-α), Interleukin-6 (IL-6) and monocyte chemoattractant protein-1 (MCP-1). Therefore, HCAR2 should be a potential target for treating dyslipidemia and inflammatory diseases.^6,9^

To date, few HCAR2 agonists niacin, acipimox, and monomethyl fumarate (MMF) have been approved. Niacin, known as vitamin B3, is used clinically to treat dyslipidemia by activating HCAR2 signaling pathways and is being investigated for the treatment of Parkinson’s disease in clinical trial (NCT03808961) recently.^10,11^ Acipimox, a niacin derivative, is used to treat hyperlipidemias.^5^ MMF, granted by FDA approval in 2020 for the treatment of multiple sclerosis, mediates beneficial effects mainly through HCAR2.^12^ However, the data from clinical trials indicated that niacin and other drugs administration would cause some side effects including headache, itching, gastrointestinal disturbance, and cutaneous flushing, limiting patient compliance.^13^ Flushing is characterized by cutaneous vasodilation accompanied by the burning sensation on face or body.^14^ HCAR2 signals mediate distinct pathophysiological events via coupling G-proteins or engaging β-arrestins. Previous study has shown that flushing is mediated by HCAR2-β-arrestin1 signaling activation even at low niacin doses.^15^ In the past decade, many efforts have been made to develop effective therapeutics with reduced side effects, like subtype-specific agonist MK-6892 and G_i_-protein biased agonist MK-0354, a potential ligand bound to the OBP of HCAR2.^16,17^

Particularly, MK-6892 is a cyclohexene carboxylic acid analog and was discovered as a high-affinity and potent selective agonist of HCAR2, displaying a significantly larger therapeutic index than niacin with reduced flushing profiles in animal model.^18^ However, the mechanisms of receptor activation and orthosteric ligand selectivity of HCAR2 remains unclear, hindering further optimization of the ligand.

Biased ligands that occupy the OBP of GPCRs were reported to achieve specific signaling pathway with therapeutic outcomes, reducing “off-target” side effects. In addition to biased orthosteric ligands, allosteric modulators of GPCR can bound to a distinct binding site and then trigger functional signaling pathway with high specificity, therefore, this type of allosteric modulators is also termed as biased allosteric modulators (BAMs).^19,20^ Different from orthosteric agonists, BAMs offer a promising strategy to control on- or off-target by occupying non-conserved binding sites in GPCRs.^21^ In particular, the allosteric modulator and agonist can bind simultaneously to GPCRs, which can achieve further functional selectivity of receptors.^22,23^ More interestingly, the specific allosteric modulator exhibits probe dependence in response to different agonists on a receptor.^24^ For instance, SBI-553, an allosteric modulator for neurotensin receptor type I (NTSR1), acts as a negative allosteric modulator (NAM) for G-protein signal pathway but as a positive allosteric modulator (PAM) for β-arrestin translocation.^25^ Another allosteric modulator LY2033298 exhibits PAM for some agonists (eg., oxotremorine or tetramethylammonium) of muscarinic acetylcholine receptor M2 mediated ERK1/2 phosphorylation pathway, but functions as a NAM for other agonists (eg., pilocarpine and xanomeline).^24^ These findings suggest that in-depth investigation of the pharmacological characteristics of an allosteric modulator is required for HCAR2. We demonstrated that the PAM compound 9n was identified to exert specific HCAR2-G_i_ protein-biased signaling in the presence of agonist niacin and to promote anti-inflammation effect in mouse model.^9^ However, the cooperativity of MK-6892 and allosteric modulator compound 9n on HCAR2 is unknown.

In this study, we investigate the orthosteric ligand selectivity among HCARs subfamily and allosteric regulation of compound 9n at HCAR2. We determined the structure of MK-6892-HCAR2-G_i_ complex using cryo-EM method, and deciphered recognition mechanism of MK-6892 with receptor. Combined with our functional assays, we further elucidated the selectivity of orthosteric ligands and the probe dependence of allosteric modulation at HCAR2. Together, our findings provide insights into understanding the pharmacological feature of HCAR2 in response to different types of ligands.

## Results

### The overall structure of MK-6892 bound HCAR2-G_i_ signaling complex

**Fig. 1:**
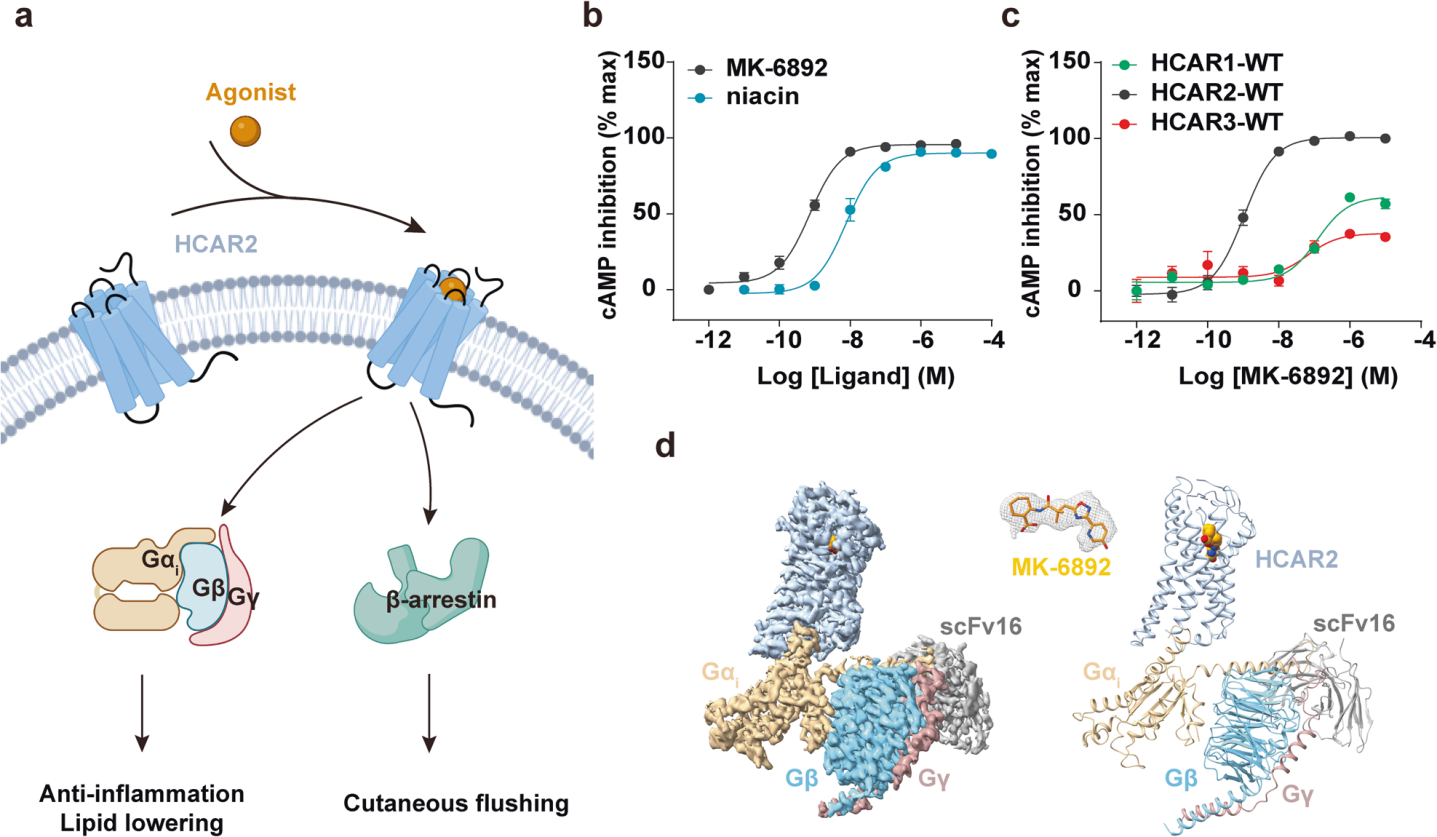
Signal transduction and structure of MK-6892-HCAR2-G_i_ complex. **a** Downstream signals and functions mediated by HCAR2. The schematic figure created with BioRender.com. **b** Represented curve for niacin and MK-6892-induced HCAR2 activation examined by cAMP inhibition assay. **c** Activating effect of MK-6892 on HCARs by cAMP inhibition assay. Data are presented as the mean ± SEM of three independent experiments performed in triplicate. **d** The cryo-EM map (left panel) and structural mode (right panel) of MK-6892-bound to HCAR2 in complex with G_i_ heterotrimer and scFv16. Orange, MK-6892; Light steel blue, HCAR2; Tan, Gα_i_; Sky blue, Gβ; Rosy brown, Gγ; Dark gray, scFv16. The EM density of HCAR2-G_i_ complex and MK-6892 was shown with the counter level of 0.62, 0.469 respectively

HCAR2 can activate G_i_ signaling as well as β-arrestin pathways, and the G_i_ activation induced by HCAR2 was demonstrated to be an important role in therapeutic outcomes (Fig. 1a).^6,15^ The side effects of orthosteric agonist like niacin might limit the clinical application in the treatment of lipid-lowering or anti-inflammatory conditions.^15^ MK-6892 is a potent agonist of HCAR2 with high selectivity. To investigate its potency and selectivity for HCAR2, we performed the forskolin-induced cAMP accumulation assay for HCARs induced by different agonists. The results reveal that MK-6892 exhibits higher G_i_-protein activation potency than niacin and has higher selectivity on HCAR2 among HCARs subfamily (Fig. 1b, c).

Understanding the binding mode and pharmacological properties of MK-6892 could accelerate to develop the next-generation agents targeting HCAR2. Here, we presented the cryo-EM structure of MK-6892-bound HCAR2 coupled with G_i_ heterotrimer (Fig. 1d). To obtain stable HCAR2-G_i_ complex, we co-expressed wild-type (WT) human HCAR2 and G-protein heterotrimer (dominant-negative Gα_i1_, Gβ_1_, and Gγ_2_). The HCAR2-G_i_ complex was assembled in the presence of MK-6892, furthermore the single-chain fragment variable antibody scFv16 was supplied to stabilize the signaling complex (Supplementary Fig. S1a). Finally, the structure of MK-6892-HCAR2-G_i_-scFv16 complex was resolved at a global resolution of 2.60 Å (Supplementary Table S1), and a clear cryo-EM density map corresponding to the compound MK-6892 was identified in the orthosteric pocket of HCAR2 (Fig. 1d and Supplementary Fig. S1). The MK-6892 pose was further validated by molecular dynamics (MD) stimulation (Supplementary Fig. S2).

The overall structure of MK-6892-bound HCAR2-G_i_ complex displays a nearly identical conformation to that of niacin-bound HCAR2 complex structure, with the root-mean-square deviation (RMSD) values of Cα being 0.6 and 0.7 for the receptor and the whole complex respectively (Supplementary Fig. S3a). Similar with niacin-bound structure, the N-terminus of HCAR2 is observed to overlay on the top of the helix bundle. Two cysteines (C18 and C19) at the N-terminus form disulfide bonds with residues C183^ECL2^ and C266 at extracellular loop 3 (ECL3) respectively (Supplementary Fig. S3b). Additionally, the residues E12 and R22 establish hydrogen bonds with the main chain of R90^ECL1^ and the side chain of N171^ECL2^ (Supplementary Fig. S3b). The extensive interactions between the N-terminus of HCAR2 and the extracellular loops (ECL1, ECL2, and ECL3) stabilize the architecture of HCAR2, which would limit ligand entry into the classic orthosteric site from the extracellular milieu.^26^ Notably, a positively charged cave formed by the extracellular proximal end of TM3, TM5 and partial N-terminus of HCAR2 may facilitate the access of orthosteric ligands bearing a negatively charged carboxyl group (Supplementary Fig. S3c). Consistently, previous studies have proposed positively charged gap between TM1 and TM7 as ligand access port in the lipid GPCRs, such as sphingosine-1-phosphate receptor 3 (S1PR3)^27^ and prostaglandin D2 receptor CRTH2^28^ (Supplementary Fig. S3c).

### Recognition mechanism of MK-6892 with HCAR2

**Fig. 2:**
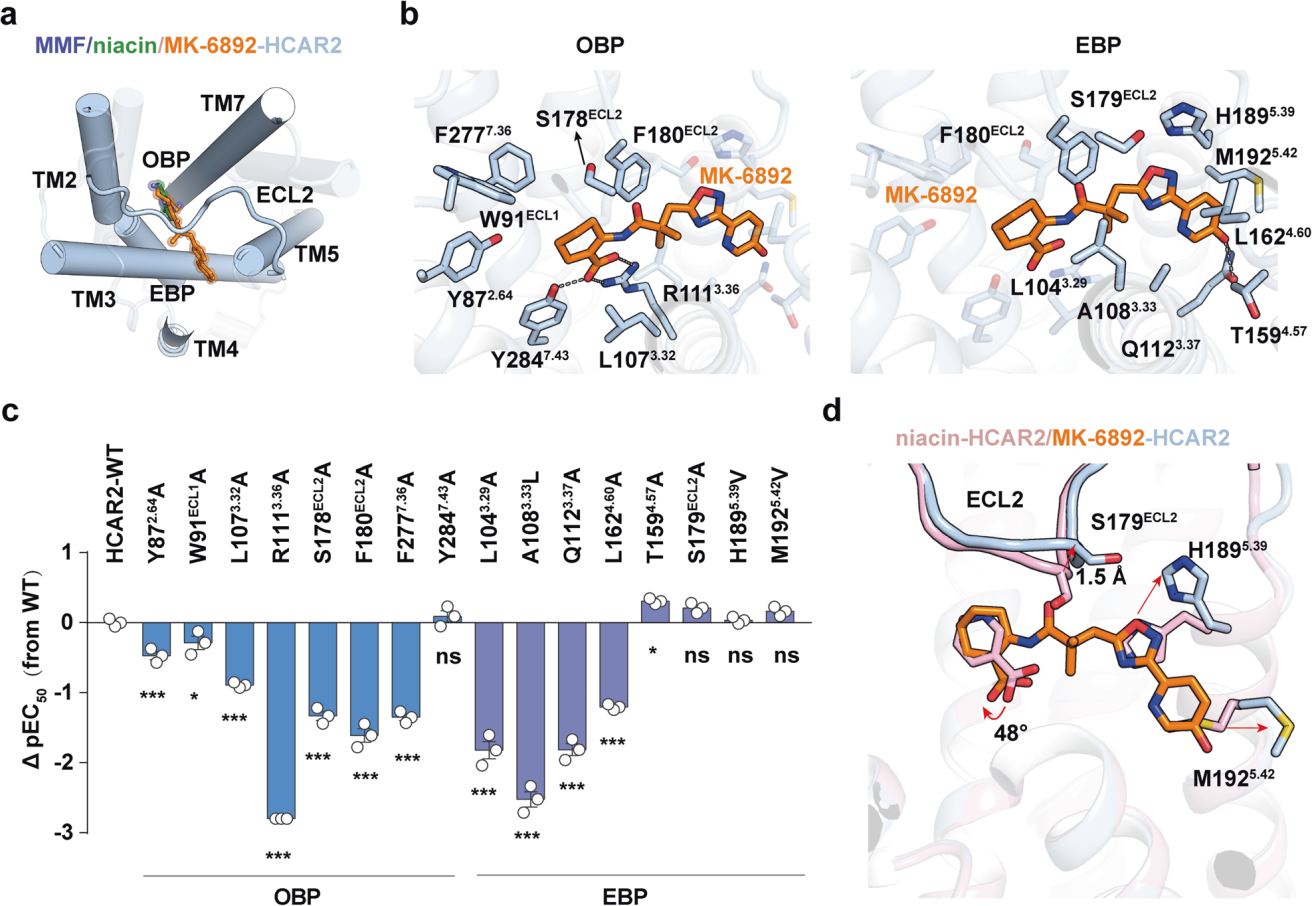
Key residues for the interaction between MK-6892 and HCAR2. **a** Structural comparison of the binding modes of niacin, MMF and MK-6892 to HCAR2. **b** The detailed interactions between MK-6892 and HCAR2 in OBP (left panel) and EBP (right panel). Black dashed lines represent polar interactions. **c** Mutagenesis effects of the residues in OBP and EBP of HCAR2 on their activities in response to niacin and MK-6892 stimulation examined by cAMP inhibition assay. The value of ΔpEC_50_ (pEC_50_MT-pEC_50_WT) shows differences between wild-type (WT) receptors and mutants (MT). Data are displayed as mean ± SEM from at least three independent experiments, each performed in triplicate. Statistical significance was determined by one-way analysis of variance with Dunnett’s multiple comparison test (compared to WT). n.s., no significance. **d** Structural comparison of the niacin- and MK-6892 bound binding pocket in HCAR2. The conformational changes between the two structures are indicated as red arrows

The structure of MK-6892-bound HCAR2 complex showed that MK-6892 assumes an inverted L shape, engaging with an enlarged binding pocket composed of TM2-TM5, TM7, and covered by ECL2. Notably, when compared with niacin or MMF bound structures, MK-6892 in HCAR2 exhibits a distinct binding mode (Fig. 2a). In detail, the binding pocket of MK-6892 in the receptor is divided into two segments by ECL2, one is defined as the OBP, which accommodates niacin or MMF as well as cyclohex-ene-1-carboxylic acid moiety of MK-6892, whereas the other is defined as the EBP, adopting 6-(1,2,4-oxadiazol-3-yl)−1,6-dihydropyridin-3-ol moiety of MK-6892 (Fig. 2b and Supplementary Fig. S4). Structural comparisons indicate that both MK-6892 and niacin do not directly contact with the microswitch residue at position 6.48 with a distance of 10.3 Å and 12.0 Å respectively, which is much larger than that in activated β2AR^29^ and CB1^30^ (Supplementary Fig. S5). It is noteworthy that the residue at position 6.48 is phenylalanine in HCAR2, but tryptophan in most common GPCRs (Supplementary Fig. S5).

Inspection of the binding pocket of HCAR2 reveals that MK-6892 makes extensive contacts with the receptor, mainly through hydrogen bonding, hydrophobic contacts, and Van der Waals forces (Fig. 2b and Supplementary Fig. S4a). In detail, the cyclohex-ene-1-carboxylic acid moiety of MK-6892 makes a salt bridge with the positively charged R111^3.36^ and forms an extra hydrogen bond with the side chain of Y284^7.43^ in OBP (Fig. 2b). In addition, the cyclohex-ene-1-carboxylic acid moiety along with dimethyl group are surrounded by a set of hydrophobic residues, including Y87^2.64^, W91^ECL1^, L107^3.32^, F180^ECL2^ and F277^7.36^ (Fig. 2b). In particular, alanine replacement of R111^3.36^ decreased the potency of G_i_ protein activation significantly due to the disruption of polar interaction, and the F180^ECL2^A mutation attenuated receptor activation remarkably, suggesting the important roles for these residues in ligand recognition (Fig. 2c and Supplementary Fig. S6, Table S2). The 6-(1,2,4-oxadiazol-3-yl)−1,6-dihydropyridin-3-ol moiety of MK-6892 resides in the hydrophobic EBP, formed by residues L104^3.29^, A108^3.33^, Q112^3.37^, L158^4.56^ and T159^4.57^ on one side, and the residues H189^5.39^, M192^5.42^, F180^ECL2^ and S179^ECL2^ on the other side (Fig. 2b). In agreement with our structural observations, these contacts were further validated by our mutagenesis studies. Mutations of L104^3.29^A, A108^3.33^L, Q112^3.37^A, and L158^4.56^A in the EBP significantly decreased the potency of HCAR2 activation induced by MK-6892 (Fig. 2c, Supplementary Fig. S6, and Table S2).

Compared with the structure of niacin-bound HCAR2, several conformational displacements are observed in the MK-6892-bound structure. One notable difference is observed in OBP, in which the cyclohex-ene-1-carboxylic acid moiety of MK-6892 in HCAR2 exhibits clockwise rotation of 48° relative to the corresponding moiety of niacin (Fig. 2d). In particular, the EBP makes notable conformational changes upon MK-6892 binding. For instance, the Cα of residue S179^ECL2^ in ECL2 is upraised by 1.5 Å to accommodate the carbonyl group of MK-6892 (Fig. 2d). The outward movement of M192^5.42^ and the upward movement of the side chain of H189^5.39^ result in the ligand-binding pocket with a larger volume (about 160 Å^3^) than that of niacin-bound HCAR2 (about 37 Å^3^; Fig. 2d and Supplementary Fig. S7). The particular EBP of HCAR2 may serve as a key element for selectivity and high efficacy of MK-6892 at HCAR2.

### The OBP determines the ligand selectivity

**Fig. 3:**
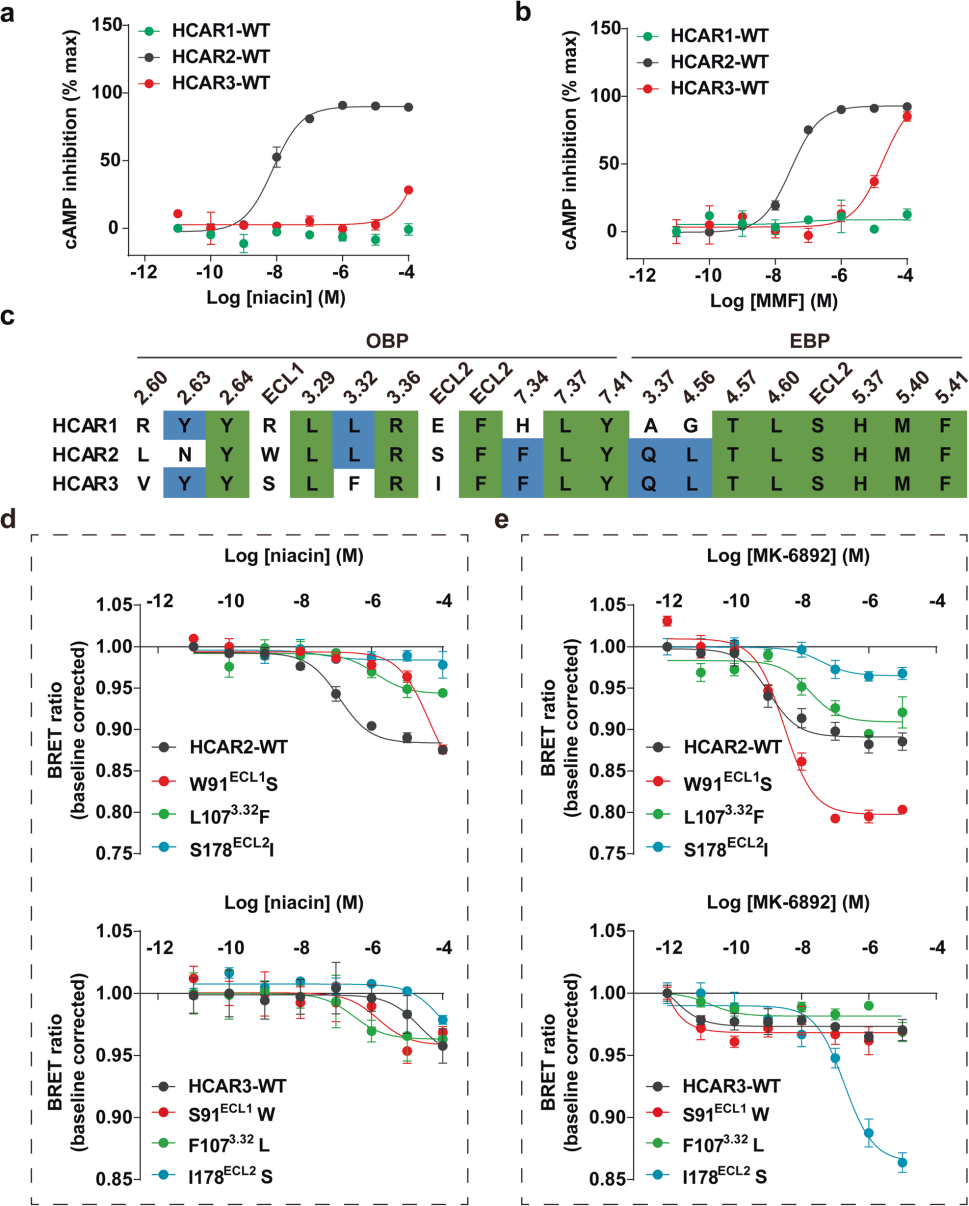
Selectivity of niacin and MK-6892 on HCARs. **a**, **b** Activation effect of niacin (**a**) and MMF (**b**) on HCARs measured by cAMP inhibition assay. Data are displayed as mean ± SEM from at least three independent experiments, each performed in triplicate. **c** Sequence alignment of the residues involved in the OBP and EBP of HCARs. Conserved residues among all the three HCARs are highlighted in green background. **d**, **e** The Gα_i1-_γ_2_ dissociation BRET assay to examine effects of niacin (**d**) and MK-6892 (**e**) on the swapped residues in HCAR2 and HACR3. Data are displayed as mean ± SEM from at least three independent experiments, each performed in triplicate

To investigate the detailed region of HCAR2 contribute to the ligand selectivity with receptor, we further performed mutagenesis studies and additional functional assays. Sequence alignment reveals that HCARs subfamily shares high similarity, especially HCAR2 and HCAR3 have approximately 95% identity.^3^ Surprisingly, the ligands (niacin, MMF and MK-6892) all display subtype-selectivity for HCAR2 (Figs. 1c and 3a, b). In our previous study, we described consensus pattern in OBP regarding niacin-, MMF-bound HCAR2 structures.^9^ Sequence alignment for the ligand (niacin and MK-6892) binding pocket reveals that several residues are not conserved in HCARs (Fig. 3c). Therefore, we substituted these residues in HCAR2 with their corresponding counterparts in HCAR3. The results of our pharmacological assays indicated that all the mutants decreased the activation potency of HCAR2 induced by niacin and MK-6892 (Fig. 3d, e). Surprisingly, the S91^ECL1^W and F107^3.32^L mutations in HCAR3 displayed increased activation potency of niacin at HCAR3 (Fig. 3d). In addition, HCAR3 I178^ECL2^S mutant restored MK-6892-induced activation (Fig. 3e). Collectively, our results indicated that the residues W91^ECL1^, L107^3.32^, and S178^ECL2^ in the OBP play critical roles in the ligand selectivity for HCAR2.

A similar swapped mutagenesis investigation was performed on HCAR1 and HCAR2. However, the replacement of the allelic residues in HCAR1 with those found in HCAR2 did not result in an increased effect of niacin or MK-6892 (Supplementary Fig. S8a, b). Conversely, the activation potency of niacin or MK-6892 increased with multipoint mutation at HCAR1 (Supplementary Fig. S8c). These results revealed that these variable residues in OBP together determined the ligands selectivity between HCAR1 and HCAR2.

### Structural basis of G_i_ protein couple with HCAR2

The structure of HCAR2-G_i_ complexes exhibit a similar global architecture with the previously reported Class A GPCR-G_i_ complexes.^31^ The engagement of G_i_ heterotrimer is mainly mediated by the interaction with TM2, TM3, TM5, TM6 and intracellular loops (ICL1, ICL2, ICL3) of HCAR2 (Supplementary Fig. S9a). Some common polar contacts have been observed to play important roles in G_i_ protein anchoring: (i) the main chain of R128^3.53^ forms a direct hydrogen bond with the side chain of N347^G.H5.19^ in Gα_i_; (ii) the side chain of R218^ICL3^ in HCAR2 makes a salt bridge with D341^G.H5.13^; and (iii) the side chain of R228^6.32^ in HCAR2 forms a polar contact with F354^G.H5.26^ (Supplementary Fig. S9b, c). Similar polar interactions have also been identified in GPCR-G_i_ complexes,^27,32,33^ suggesting that these interactions play determinant roles in G_i_ protein coupling.

HCAR2 belongs to a family of receptors that respond metabolic short-chain fatty acids (hydroxycarboxylic acid), we thus compared the structure of HCAR2 with available structures of medium or long-chain fatty acid sensing receptors GPR84 and GPR120, a notable conformation displacement of α5 helix of Gα_i_ was observed in HCAR2 (Supplementary Fig. S9d).^34,35^ The short TM5 in HCAR2 could decrease contacts with the Ras-like domain and α5 helix of Gα_i_ protein, resulting in direct interactions of α5 helix with ICL1 and ICL2 in HCAR2 (Supplementary Fig. S9e). In detail, the residue R63^ICL1^ in HCAR2 makes hydrogen bonding with the main chain of C351^G.H5.23^ in Gα_i_ (Supplementary Fig. S9f) and the side chain of H133^ICL2^ inserts into a hydrophobic cavity composed by L194^G.S3.01^,V339^G.H5.11^, T340^G.H5.12^, and I343^G.H5.15^ of Gα_i_ (Supplementary Fig. S9g).

### Probe dependence of allosteric modulator compound 9n

Allosteric modulators are a promising strategy to achieve subtype selectivity and specific signaling pathway. We first demonstrated that compound 9n of HCAR2 is a biased allosteric modulator that prefers G_i_-protein signaling.^9^ To further understand the pharmacologic characteristics of compound 9n, we next investigated the cooperativity effects of compound 9n and different agonists at HCAR2 signaling pathways.

**Fig. 4:**
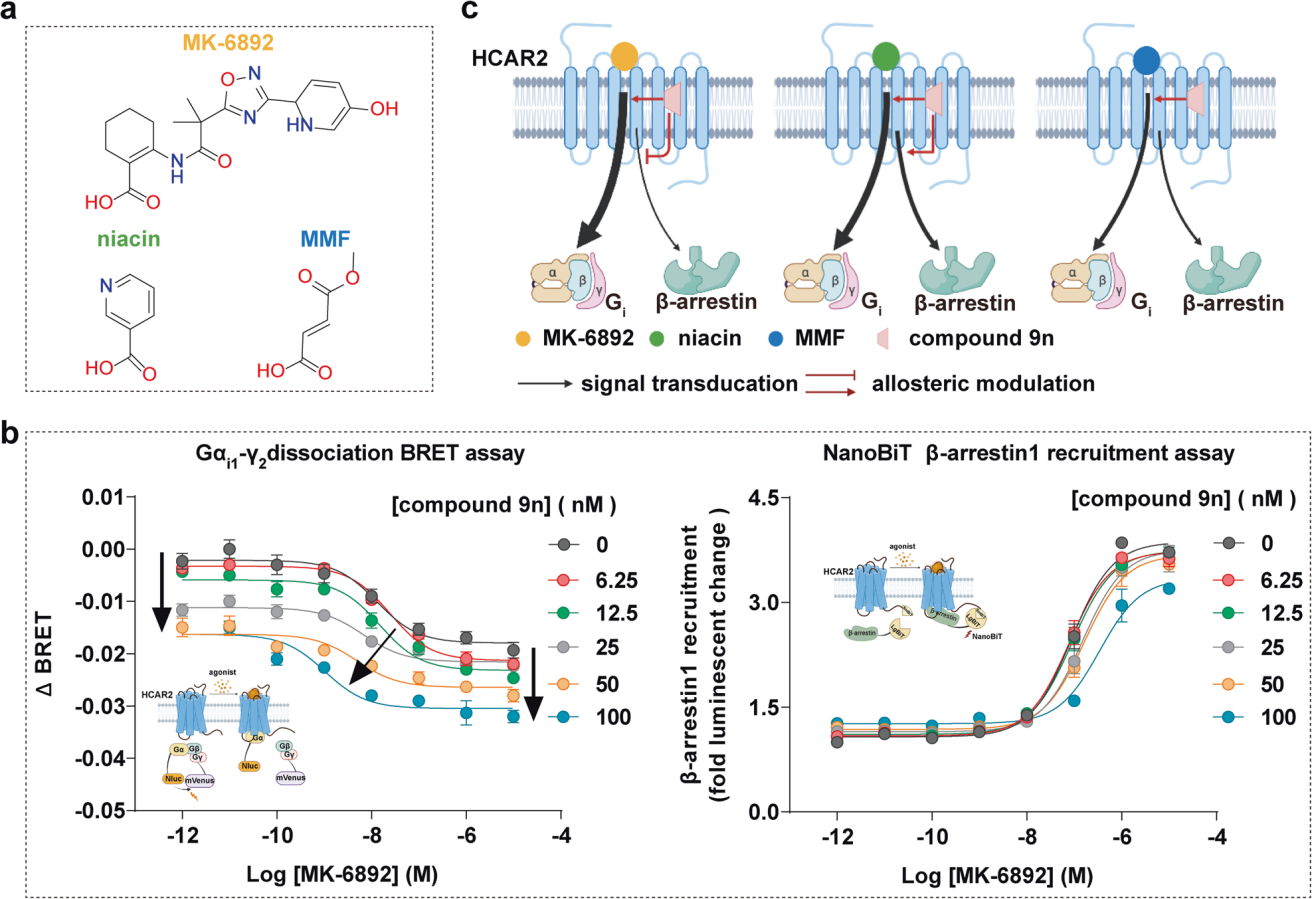
The allosteric effect of compound 9n on HCAR2 downstream signaling induced by MK-6892. **a** The chemical structures of orthosteric agonists on HCAR2. **b** The allosteric effect of compound 9n on HCAR2 downstream signaling induced by MK-6892. The G_i_ protein signaling was determined by BRET-based G-protein dissociation assay (left panel) and β-arrestin1 recruitment was examined by NanoBiT assay (right panel). Dose-dependent curves were shown. Data are displayed as mean ± SEM from at least three independent experiments, each performed in triplicate. **c** Schematic presentation of the allosteric effect of compound 9n with the different orthosteric agonists at HCAR2. The figure created with BioRender.com

MK-6892 has distinct chemical structures with niacin or MMF, exhibiting a different binding pose in HCAR2 (Fig. 4a). Compared with the potencies of niacin or MMF, MK-6892 displayed approximately 10-40-fold increase on HCAR2 mediated G_i_ protein signaling response. Compound 9n was proved to increase binding affinity and activation efficacy of niacin.^16,36^ Consistently, we found that compound 9n exhibited PAM efficacy on MMF.^9^ Interestingly, the results of bioluminescence resonance energy transfer (BRET)-based Gα_i1_-Gγ_2_ dissociation assay and cAMP inhibition assay further revealed that compound 9n potentiated the MK-6892-induced G_i_ protein activation in a dose-dependent manner (Fig. 4b and Supplementary Fig. S10), suggesting that compound 9n exerts positive cooperativity with MK-6892 as well as HCAR2-G_i_ signaling preference.

By contrast, for β-arrestin recruitment, compound 9n displayed a subtle PAM effect towards niacin or a neutral allosteric ligand (NAL) for MMF, the compound 9n acted as a negative allosteric modulator (or antagonist) for HCAR2-β-arrestin signaling by MK-6892 in a dose-dependent manner (Fig. 4b, c). These phenomena suggest the pharmacological characteristic of compound 9n depends on different orthosteric ligands of HCAR2, representing a probe dependence manner (Fig. 4c). More importantly, previous studies have shown that the β-arrestin pathway of HCAR2 mediates the adverse effect of skin flushing, whereas G-protein signaling contributes to lipolysis and the treatment of inflammatory.^6,15^ Therefore, probe dependence would tell us how to consider a choice of orthosteric ligand to evaluate the efficacy of allosteric modulator. Our results suggest that combination treatment of compound 9n with MK-6892 could be a therapeutic strategy against inflammation and lipid-lowering.

## Discussion

GPCRs regulate the physiological processes via sensing to the endogenous ligands. Generally, each endogenous ligand could activate multiple receptor subtypes, which contains conserved orthosteric binding pocket. Severe pathological side effects related to off-target pharmacological activities. Development of a selective ligand target specific receptor subtype with desired therapeutic is still challenge in GPCR drug discovery field. The dynamic and plasticity of ligand binding pocket in GPCR offer opportunities to identify new druggable site (EBP), for example, dopamine receptor contains five subtypes (DRD1-DRD5), the subtype-selective ligands SKF83959 and PW0464, which target DRD1, bind to the EBP formed by the extracellular regions of TM2-3 and TM6-7 and exhibit high affinity for DRD1.^37^ In contrast, the antagonist haloperidol prefers to interact with a distinct EBP, comprised of TM2-3 and ECL1-2, and this specific recognition confers haloperidol’s high selectivity for DRD2, as compared to its affinity for DRD3 and DRD4.^38^

HCAR2 represents a promising therapeutic target for the treatment of cardiovascular and neurological disorders, owing to its anti-lipolytic and anti-inflammatory functions.^10,13,15^ Approved agonists of HCAR2, including niacin, its derivative acipimox, and MMF, are utilized for the treatment of dyslipidemia, hyperlipidemias, and multiple sclerosis.^8,18,22^ Nonetheless, these drugs are associated with an undesirable side effect: cutaneous flushing, which limits the broader application of HCAR2-targeted medications.^8,18,22–24^ To address this issue, several selective ligands, such as MK-6892 and GSK256073, have been designed with distinct chemical structures that diverge from niacin.^27,29,39^ These novel ligands have demonstrated a significant reduction in flushing profiles within animal models. However, the similarities and differences in the recognition models between approved drugs and subtype-selective ligands, such as MK-6892, largely unexplored.

In our study, we determined a high-resolution structure of HCAR2-G_i_ signaling complex bound to the selective agonist MK-6892. Combined with the niacin- or MMF-bound HCAR2 structures resolved in our previous research,^9^ the N-terminus and extracellular loops of HCAR2 form a special gap, which appears to restrict ligand entry from the extracellular milieu. We speculated a potential entry pathway for ligands situated at the extracellular proximal end of TM3 and TM5. However, this proposed entryway remains speculative and warrants further experimental verification. In the binding pocket, the core moiety of MK-6892, niacin and MMF all share a similar binding pose and adopt a same interaction with residues R111^3.36^, F180^ECL2^, and Y284^7.43^ in the OBP of HCAR2. Among them, R111^3.36^, forms a salt bridge with the carboxyl group of ligands, serving as an anchoring site and considered a key residue for ligands recognition and receptor activation. Compared with niacin- and MMF-bound HCAR2, MK-6892 extends into the EBP and forms an additional hydrogen bond with Q112^3.37^, explaining the reason for the higher affinity of MK-6892. We suppose that the higher activation potency for HCAR2 induced by MK-6892 may provide a broad therapeutic window while limiting the activation of the cutaneous flushing pathway. Furthermore, together with mutagenesis results, we found that the key residues in OBP play a critical role for the selectivity of MK-6892 among HCARs subfamily, and the ligands (niacin and MMF) also display subtype-selectivity for HCAR2.

In addition to the selectivity of orthosteric ligands, functional selectivity, also known as biased signaling, this phenomenon describes a ligand’s ability to preferentially activate one of several signaling pathways mediated with a receptor.^40–43^ GPCRs can engage with diverse signaling pathways by coupling with different transducers, including diverse G-proteins (G_s_, G_i/o_, G_11/q_ and G_12/13_) and β-arrestins (β-arrestin1 and β-arrestin2), each of which can mediate distinct physiological functions. A significant challenge in drug development arises when the activation of non-therapeutic signaling pathways, stemming from a drug targeting a specific receptor, leads to undesirable on-target side effects. Biased ligands emerge as a potential solution to this issue. These ligands are designed to selectively activate a desired signaling pathway while concurrently attenuating or entirely inhibiting others, thereby substantially reducing the risk of on-target side effects. For instance, oliceridine acts as a G-protein-biased agonist for the μ-opioid receptor (μOR).^44^ By selectively activating the G_i_ signaling pathway, oliceridine aims to provide effective pain relief while reducing common side effects, such as respiratory depression and constipation, that are often associated with β-arrestin pathway activation.

HCAR2 could meditate the G_i/o_ and β-arrestins signal pathways induced by niacin, MMF and MK-6892. Previous studies indicated that the side effects caused by niacin was related with the β-arrestin-dependent signaling pathway.^15^ Our previous study also suggests that the β-arrestin signaling pathway is not involved in anti-inflammatory processes. Allosteric modulators, especially BAMs, not only have receptor subtype selectivity but also have the ability to control receptor signaling pathways.^19,45^ In our previous study, we have identified that the compound 9n performed as a G_i_-biased allosteric modulator for HCAR2 and enhanced the anti-inflammatory effects in mouse model of colitis when used alongside niacin.^9^ However, the modulation effect of compound 9n with subtype-specific MK-6892 was still unknown. In the present study, we conducted a thorough investigation into pharmacologic characteristics of allosteric modulator compound 9n. For G_i_ signal pathway of HCAR2, compound 9n functions not only as a PAM for HCAR2, but also exhibits a unique property (probe dependence) in its allosteric modulation. The cooperativity of compound 9n with agonists demonstrates varying efficacies that depends on the activation potencies of agonists. Specifically, the higher the efficacy of the orthosteric agonist (e.g., MK-6892), the more pronounced the PAM effect induced by compound 9n. Remarkably, for β-arrestin signal pathway, compound 9n exhibits a different behavior. Instead of displaying a PAM effect towards niacin or a NAL effect for MMF, compound 9n could antagonize the β-arrestin signaling of HCAR2 that is induced by MK-6892. This pharmacological action suggests a potential co-administration strategy: pairing MK-6892 with compound 9n could potentially increase the therapeutic window for disease treatment, thereby enhancing clinical outcomes. Further animal model experiments or clinical trials are needed to estimate the therapeutic efficacy of this combination.

Together, our study provides insights into ligand recognition and activation regulation of HCAR2, as well as investigates pharmacological characteristics of allosteric modulator compound 9n on receptor signaling. These insights hold the potential to significantly guide the development of drugs targeting HCAR2, aiming for enhanced efficacy and minimized side effects, whether off-target or on-target. Furthermore, this study offers an opportunity to understand the translation of the combination of agonist and allosteric modulator in the future and the complex pharmacological features of allosteric modulators.

## Materials and methods

### Generation of HCAR2 constructs

The expression construct of human HCAR2, followed with a haemagglutinin (HA) signal sequence, Flag tag at the N-terminus, was inserted into the pFastBac1 (Invitrogen) baculovirus expression vector. The human DNGα_i1_ (S47N, G203A, E245A, A326S) and human Gβ_1_, bovine Gγ_2_ was constructed into pFastBac1(Invitrogen) and pFastBac-dual (Invitrogen) vector respectively.

### Generation of scFv16

The antibody scFv16 with a GP67 signal peptide at the N-terminus and 6 × His tag at the C-terminus was inserted into pFastBac1 vector and expressed by the *Sf9* baculovirus system. The Ni-NTA resin, followed by a molecular exclusion chromatography column (Superdex 75 Increase 10/300 GL, Cytiva) was be used to obtain the antibody scFv16 which was further concentrated to 4 mg/mL. Finally, the protein was flash frozen in liquid nitrogen and stored at −80 °C for usage in MK-6892-bound HCAR2-G_i_ complex purification.

### Expression and purification of HCAR2-G_i1_ complex

*Sf9* cells were cultured in ESF 921 medium (Expression Systems) at a density of 2.8 × 10^6^ cells/mL, then co-infected with baculoviruses containing HA-Flag-HCAR2, DNGα_i1_ and Gβ_1_γ_2_ at a ratio of 1:2:1. After 48 h of infection, cells were harvested and resuspended with buffer (20 mM HEPES, pH 7.5, 50 mM NaCl, 5 mM CaCl_2_, 5 mM MgCl_2_) supplemented with protease inhibitors (100 μg/mL leupeptin, 160 μg/mL benzamidine). The HCAR2-G_i1_ complex was formed on the membrane by adding 10 µM MK-6892 and 25 mU/mL apyrase (NEB) for 2 h at 25 °C and solubilized by 0.1% (w/v) cholesteryl hemisuccinate (CHS), 0.5% (w/v) lauryl maltose neopentyl glycol (LMNG; Anatrace) at 4 °C for 2 h. The antibody scFv16 was supplement to stable the complex. After centrifugated at 65,000×*g* for 30 min, we transferred the supernatant to the tubes and incubated with the M1 anti-Flag resin (Sigma-Aldrich) at 4 °C for 2 h. The mixture was loaded into a gravity column, and washed with 30 column volumes of the washing buffer (20 mM HEPES, pH 7.5, 100 mM NaCl, 5 mM CaCl_2_, 0.01% (w/v) LMNG, 0.001% (w/v) CHS, 10 μM MK-6892, the protease inhibitors). The complex was collected with the washing buffer supplemented with 10 mM EDTA, 0.2 mg/mL Flag peptide, concentrated by concentrator (100 kDa molecular-weight cut-off, Millipore) and then loaded onto a molecular exclusion chromatography column (Superdex 200 10/300 Increase GL column, Cytiva) with the buffer (20 mM HEPES, pH 7.5, 100 mM NaCl, 0.00075% (w/v) LMNG, 0.0002% (w/v) CHS, 0.00025% glyco-diosgenin (GDN; Anatrace) and 10 µM MK-6892. The purified complex fractions were collected and concentrated to 16.8 mg/mL for usage.

### Cryo-grid preparation and EM data collection

The purified MK-6892-bound HCAR2-G_i_-scFv16 complex (3 µL) were loaded onto 300-mesh Au holey carbon grids (Quantifoil R1.2/1.3), which were glow-discharged at 15 mA for 60 s before use. The Vitrobot Mark IV (Thermo Fisher) was set at 4 °C and 100% humidity for blotting. The grids were blotted for 2-3 s and subsequently immersed in liquid ethane. The grids with MK-6892-bound HCAR2-G_i_-scFv16 complex were applied into a Titan Krios cryo-electron microscope (Thermo Fisher), the data were automatically collected in a K3 direct electron detector (Gatan) at 300 keV with the magnification of 130,000 by using SerialEM^46^ software. All the movie stacks were recorded in super-resolution mode at a dose rate of 20 e^-^/pix/s with a total exposure time of 2.7 s, resulting in a total dose of 65 e^−^/Å^2^ per stack including 40 frames.

### Image processing and 3D reconstructions

The collected 5676 movies were first processed by Motioncor2^47^ and Gctf^48^ successively. Subsequently, 5,157,653 particles in total were picked in Relion 4.0.^49^ Then the particles were sorted by 2D and 3D classification, and one 3D class with good shape were extracted and imported into cryoSPARC3.1.^50^ These particles were heterogeneous refined with four 3D references from 3D classification as templates, a sharp class containing 645,569 particles was subjected to non-uniform refinement, producing a sharpened map with an overall resolution of 2.83 Å. The refined particles were clustered using 3D variability analysis, the homogenous 511,734 particles were further refined to 2.60 Å resolution which was estimated by the Fourier shell correlation with 0.143 criterion. Local refinement focusing on the G_i_ proteins and HCAR2 generated a 2.62 and 2.87 Å map, respectively. The map for receptor and G_i_ protein were combined in UCSF Chimera^51^ using ‘vop maximum’ command. The local resolution was evaluated in cryoSPARC3.1.

### Model building and structure refinement

The initial model of MK-6892-bound HCAR2-G_i_ complex was obtained from the structure of niacin-HCAR2-G_i_ complex reported in our previous research.^9^ The complex model with niacin removed was fitted and placed in the corresponding EM density map by UCSF Chimera,^51^ then the agonist MK-6892 was added and manual adjusted in Coot,^52^ real-space refinement were performed in Phenix^53^ with several rounds subsequently. MolProbity^54^ was used to validate the final model. UCSF ChimeraX^55^ and PyMOL (https://pymol.org/2/) software were used for preparing the structural figures.

### Molecular dynamics simulation

The initial model of HCAR2-MK-6892 for MD simulation was obtained from this paper and oriented by running PPM 2.0.^56^ The MD simulation system was generated using the CHARMM-GUI web interface^57,58^ and carried out on the GROMACS 2019.6,^59^ with ff14SB,^60^ lipid21,^61^ and gaff2^62^ force field for protein, lipids, and ligand respectively, along with the TIP3 water model. The model was embedded into palmitoyl-oleoyl-phosphatidylcholine (POPC) lipid bilayers in a regular hexagonal prism box with a side length about 7.5 nm × 7.5 nm × 10.5 nm. An ion concentration of 150 mM NaCl was added, and the system charge was neutralized by replacing water molecules with ions.

Each atom was assigned a velocity randomly and independently. The steepest descent algorithm was used for minimizing system energy. For equilibrate MD simulation system, NVT ensemble for 250 ps with a time step of 1 fs and NPT ensemble at 310.15 K and 1 bar were performed. The final system was subjected to a simulation for 300,000 ps at 310.15 K and 1 bar. The gmx_rms utility was used to analyze the RMSD of the trajectory.

### cAMP inhibition assay

The GloSensor cAMP assay was used to measure activating effects of HCAR2 and its mutations referring to the previous research.^63^ Firstly, pcDNA3.1-HCAR2 or its mutations with GloSensor reporter plasmids were co-transfected into a six-well plates confluent with HEK293 cells. After 24 h transfection, the cells were re-plated in 96-well plates with the Hank’s Balanced Salt Solution (HBSS) buffer containing d-Luciferin-Potassium Salt (YEASEN). Then the cells were stimulated with the corresponding agonist with adding 5 μM forskolin at the same time. To determine the allosteric regulation efficacy, different concentrations of compound 9n were added additionally. Finally, Synergy H1 microplate reader (BioTek) was used to read the luminescence. Data were fitted in GraphPad Prism 9 by using the nonlinear regression (curve fit) dose-response function.

### NanoBiT β-arrestin assay

The HCAR2 β-arrestin signaling induced by ligand was detected by the NanoBiT β-arrestin recruitment assays. The β-arrestin1 fused the LgBiT at N-terminus was co-transfected into HEK293 cells in the 6-well plates with equal proportions of HCAR2 followed by the SmBiT at C-terminus. After 24 h of transfection, the cells were dispensed in a 96-well plate, cultured at 37 °C for 12 h, and then washed with Phosphate-Buffered Saline (PBS) buffer for once, incubated in HBSS buffer supplemented with 5 μM coelenterazine h (YEASEN) for 30 min. The baseline luminescent and ligand-induced luminescent changes were measured by the microplate reader (Synergy H1, BioTek). To determine the allosteric regulation efficacy of different ligands on HCAR2, different concentrations of compound 9n were added additionally. We analyzed the data in GraphPad Prism 9 by using the nonlinear regression (curve fit) dose-response function.

### BRET dissociation assay

We used Gα_i1_-Gγ_2_ dissociation assay to explore the activation of HCARs induced by agonists according to previous publications.^30^ Assay were performed in HEK293 cells, which transiently transfected with various versions of HCARs and BRET sensors including Gα_i_-Nluc, Gβ, and Gγ-mVenus plasmids. 24 h after transfection, cells were resuspended in complete fresh medium and plated in 96-well plates. The next day, medium was changed and replaced with BRET assay buffer supplemented with final concentration of 5 µM coelenterazine h. Next, the diluted agonists were added into wells before reading the plate at 37 °C in a microplate reader (Synergy H1, BioTek). ΔBRET was calculated by subtracting the vehicle-treated wells from the ligand-treated wells. The other way of analysis was that the BRET ratio of ligand-treated wells was divided by the vehicle control. All data points were fitted using a simulation dose-response function model in Prism 9.

### Enzyme-linked immunosorbent assay

Enzyme-linked immunosorbent assay was performed to measure the cell surface expression level of HCAR2 wild-type or mutants. The plasmids or empty pcDNA3.1 (+) vector (as a negative control) were co-transfected into a six-well plate confluent with HEK293 cells respectively. After cultured in 37 °C incubator for 24 h, digested cells were re-plated on pre-coated 96-well plates with poly-d-lysine using fresh medium. The next day, we used 5% (w/v) BSA to block cells after 15 min incubation with 4% (w/v) paraformaldehyde. Hereafter, anti-Flag HRP conjugated monoclonal antibody (1:2000 dilution) was used to treat cells overnight. Then the plates were softly washed with PBS buffer three times and added with HRP substrate 3,3′,5,5′-tetramethylbenzidine (TMB). The wells were added an equal volume of 0.5 M HCl solution to quench the reactions. The absorbance values were counted at 450 nm using the microplate reader (Synergy H1, BioTek) and normalized to the wild-type HCAR2 and graphed as a percentage of wild-type using GraphPad Prism 9.

### Supplementary information

Supplement for MK-6892-HCAR2_clear

## Data Availability

All data produced or analyzed in our study is included in the main text or the supplementary materials. The cryo-EM density map and atomic coordinate of MK-6892-HCAR2-G_i_ complex have been deposited in the Electron Microscopy Data Bank (EMDB) and Protein Data Bank (PDB) under accession numbers EMD-36736 (composite map), EMD-36737 (consensus map), EMD-36738 (receptor map), EMD-36739 (G_i_ protein map) and 8JZ7, respectively.

## References

[1] Venkatakrishnan AJ (2013). Molecular signatures of G-protein-coupled receptors. Nature.

[2] Neubig RR, Siderovski DP (2002). Regulators of G-protein signalling as new central nervous system drug targets. Nat. Rev. Drug Discov..

[3] Offermanns S (2011). International union of basic and clinical pharmacology. LXXXII: nomenclature and classification of hydroxy-carboxylic acid receptors (GPR81, GPR109A, and GPR109B). Pharmacol. Rev..

[4] Blad CC, Ahmed K, IJzerman AP, Offermanns S (2011). Biological and pharmacological roles of HCA receptors. Adv. Pharmacol..

[5] Soga T (2003). Molecular identification of nicotinic acid receptor. Biochem. Biophys. Res. Commun..

[6] Li Z, McCafferty KJ, Judd RL (2021). Role of HCA(2) in regulating intestinal homeostasis and suppressing colon carcinogenesis. Front. Immunol..

[7] Carlson LA, Oro L (1962). The effect of nicotinic acid on the plasma free fatty acid; demonstration of a metabolic type of sympathicolysis. Acta Med. Scand..

[8] Parodi B (2015). Fumarates modulate microglia activation through a novel HCAR2 signaling pathway and rescue synaptic dysregulation in inflamed CNS. Acta Neuropathol..

[9] Zhao, C. et al. Biased allosteric activation of ketone body receptor HCAR2 suppresses inflammation. *Mol. Cell* S1097-2765, 00605-6 (2023). 10.1016/j.molcel.2023.07.030 37597514

[10] Vosper H (2009). Niacin: a re-emerging pharmaceutical for the treatment of dyslipidaemia. Br. J. Pharmacol..

[11] Chong R (2021). Niacin enhancement for Parkinson’s disease: an effectiveness trial. Front. Aging Neurosci..

[12] Parodi B, Sanna A, Cedola A, Uccelli A, Kerlero de Rosbo N (2021). Hydroxycarboxylic Acid Receptor 2, a Pleiotropically Linked Receptor for the Multiple Sclerosis Drug, Monomethyl Fumarate. Possible Implications for the Inflammatory Response. Front. Immunol..

[13] MacKay D, Hathcock J, Guarneri E (2012). Niacin: chemical forms, bioavailability, and health effects. Nutr. Rev..

[14] Davidson MH (2008). Niacin use and cutaneous flushing: mechanisms and strategies for prevention. Am. J. Cardiol..

[15] Walters RW (2009). beta-Arrestin1 mediates nicotinic acid-induced flushing, but not its antilipolytic effect, in mice. J. Clin. Invest..

[16] Shen HC (2008). Discovery of pyrazolopyrimidines as the first class of allosteric agonists for the high affinity nicotinic acid receptor GPR109A. Bioorg. Med. Chem. Lett..

[17] Semple G (2008). 3-(1H-tetrazol-5-yl)-1,4,5,6-tetrahydro-cyclopentapyrazole (MK-0354): a partial agonist of the nicotinic acid receptor, G-protein coupled receptor 109a, with antilipolytic but no vasodilatory activity in mice. J. Med. Chem..

[18] Shen HC (2010). Discovery of a biaryl cyclohexene carboxylic acid (MK-6892): a potent and selective high affinity niacin receptor full agonist with reduced flushing profiles in animals as a preclinical candidate. J. Med. Chem..

[19] Slosky LM, Caron MG, Barak LS (2021). Biased allosteric modulators: new frontiers in GPCR drug discovery. Trends Pharmacol. Sci..

[20] Shen S (2023). Allosteric modulation of G protein-coupled receptor signaling. Front. Endocrinol. (Lausanne).

[21] Fu Y (2021). Cartilage oligomeric matrix protein is an endogenous beta-arrestin-2-selective allosteric modulator of AT1 receptor counteracting vascular injury. Cell Res..

[22] Kenakin T, Miller LJ (2010). Seven transmembrane receptors as shapeshifting proteins: the impact of allosteric modulation and functional selectivity on new drug discovery. Pharmacol. Rev..

[23] Keov P, Sexton PM, Christopoulos A (2011). Allosteric modulation of G protein-coupled receptors: a pharmacological perspective. Neuropharmacology.

[24] Valant C, Felder CC, Sexton PM, Christopoulos A (2012). Probe dependence in the allosteric modulation of a G protein-coupled receptor: implications for detection and validation of allosteric ligand effects. Mol. Pharmacol..

[25] Krumm BE (2023). Neurotensin receptor allosterism revealed in complex with a biased allosteric modulator. Biochemistry.

[26] Shao Z (2016). High-resolution crystal structure of the human CB1 cannabinoid receptor. Nature.

[27] Zhao C (2022). Structural insights into sphingosine-1-phosphate recognition and ligand selectivity of S1PR3-Gi signaling complexes. Cell Res.

[28] Wang L (2018). Structures of the human PGD2 receptor CRTH2 reveal novel mechanisms for ligand recognition. Mol. Cell.

[29] Rasmussen SG (2011). Crystal structure of the beta2 adrenergic receptor-Gs protein complex. Nature.

[30] Yang X (2022). Molecular mechanism of allosteric modulation for the cannabinoid receptor CB1. Nat. Chem. Biol..

[31] Yin J (2020). Structure of a D2 dopamine receptor-G-protein complex in a lipid membrane. Nature.

[32] Krishna Kumar K (2019). Structure of a signaling cannabinoid receptor 1-G protein complex. Cell.

[33] Xu P (2021). Structural insights into the lipid and ligand regulation of serotonin receptors. Nature.

[34] Mao, C. et al. Unsaturated bond recognition leads to biased signal in a fatty acid receptor. *Science* **380**, eadd6220 (2023). 10.1126/science.add6220 36862765

[35] Liu H (2023). Structural insights into ligand recognition and activation of the medium-chain fatty acid-sensing receptor GPR84. Nat. Commun..

[36] Blad CC (2012). Novel 3,6,7-substituted pyrazolopyrimidines as positive allosteric modulators for the hydroxycarboxylic acid receptor 2 (GPR109A). J. Med. Chem..

[37] Xiao P (2021). Ligand recognition and allosteric regulation of DRD1-Gs signaling complexes. Cell.

[38] Fan L (2020). Haloperidol bound D(2) dopamine receptor structure inspired the discovery of subtype selective ligands. Nat. Commun..

[39] Sprecher D (2015). Discovery and characterization of GSK256073, a non-flushing hydroxy-carboxylic acid receptor 2 (HCA2) agonist. Eur. J. Pharmacol..

[40] Kenakin T, Christopoulos A (2013). Signalling bias in new drug discovery: detection, quantification and therapeutic impact. Nat. Rev. Drug Discov..

[41] Wang W, Qiao Y, Li Z (2018). New insights into modes of GPCR activation. Trends Pharmacol. Sci..

[42] Zhuang Y (2022). Molecular recognition of morphine and fentanyl by the human mu-opioid receptor. Cell.

[43] Faouzi A (2023). Structure-based design of bitopic ligands for the micro-opioid receptor. Nature.

[44] Soergel DG (2014). Biased agonism of the mu-opioid receptor by TRV130 increases analgesia and reduces on-target adverse effects versus morphine: A randomized, double-blind, placebo-controlled, crossover study in healthy volunteers. Pain.

[45] Leach K, Sexton PM, Christopoulos A (2007). Allosteric GPCR modulators: taking advantage of permissive receptor pharmacology. Trends Pharmacol. Sci..

[46] Mastronarde DN (2003). SerialEM: a program for automated tilt series acquisition on tecnai microscopes using prediction of specimen position. Microsc. Microanal..

[47] Zheng SQ (2017). MotionCor2: anisotropic correction of beam-induced motion for improved cryo-electron microscopy. Nat. Methods.

[48] Zhang K (2016). Gctf: Real-time CTF determination and correction. J. Struct. Biol..

[49] Kimanius D, Dong L, Sharov G, Nakane T, Scheres SHW (2021). New tools for automated cryo-EM single-particle analysis in RELION-4.0. Biochem. J..

[50] Punjani A, Rubinstein JL, Fleet DJ, Brubaker MA (2017). cryoSPARC: algorithms for rapid unsupervised cryo-EM structure determination. Nat. Methods.

[51] Pettersen EF (2004). UCSF Chimera–a visualization system for exploratory research and analysis. J. Comput. Chem..

[52] Emsley P, Cowtan K (2004). Coot: model-building tools for molecular graphics. Acta Crystallogr. D Biol. Crystallogr..

[53] Adams PD (2010). PHENIX: a comprehensive Python-based system for macromolecular structure solution. Acta Crystallogr. D Biol. Crystallogr..

[54] Chen VB (2010). MolProbity: all-atom structure validation for macromolecular crystallography. Acta Crystallogr. D Biol. Crystallogr..

[55] Pettersen EF (2021). UCSF ChimeraX: Structure visualization for researchers, educators, and developers. Protein Sci..

[56] Lomize MA, Pogozheva ID, Joo H, Mosberg HI, Lomize AL (2012). OPM database and PPM web server: resources for positioning of proteins in membranes. Nucleic Acids Res..

[57] Jo S, Kim T, Iyer VG, Im W (2008). CHARMM-GUI: a web-based graphical user interface for CHARMM. J. Comput. Chem..

[58] Brooks BR (2009). CHARMM: the biomolecular simulation program. J. Comput. Chem..

[59] Pall S (2020). Heterogeneous parallelization and acceleration of molecular dynamics simulations in GROMACS. J. Chem. Phys..

[60] Maier JA (2015). ff14SB: improving the accuracy of protein side chain and backbone parameters from ff99SB. J. Chem. Theory Comput..

[61] Dickson CJ, Walker RC, Gould IR (2022). Lipid21: complex lipid membrane simulations with AMBER. J. Chem. Theory Comput..

[62] He X, Man VH, Yang W, Lee TS, Wang J (2020). A fast and high-quality charge model for the next generation general AMBER force field. J. Chem. Phys..

[63] Ping YQ (2021). Structures of the glucocorticoid-bound adhesion receptor GPR97-G(o) complex. Nature.

