# Supplementary material for: Orthosteric ligand selectivity and allosteric probe dependence at Hydroxycarboxylic acid receptor HCAR2
Source: Signal Transduct Target Ther. 2023 Sep 25;8:364. doi: 10.1038/s41392-023-01625-y (PMC10518311; doi:10.1038/s41392-023-01625-y)
Supplement: Supplementary file 1 — Supplement for MK-6892-HCAR2_clear [file 41392_2023_1625_MOESM1_ESM.docx]

Supplementary Materials for

**Orthosteric ligand selectivity and allosteric probe dependence at Hydroxycarboxylic acid receptor HCAR2**

Lin Cheng^1#^, Suyue Sun^2#^, Heli Wang^2#^, Chang Zhao^2#^, Xiaowen Tian^2#^, Ying Liu^2^, Ping Fu^2^, Zhenhua Shao^2, 3*^, Renjie Chai^1, 4, 5*^ and Wei Yan^2*^

Correspondence to: zhenhuashao@scu.edu.cn (Z.S.); renjiec@seu.edu.cn (R.C.); weiyan2018@scu.edu.cn (W.Y.).

**This PDF file includes:**

Supplementary Fig. S1 to S10

Supplementary Table S1 to S2


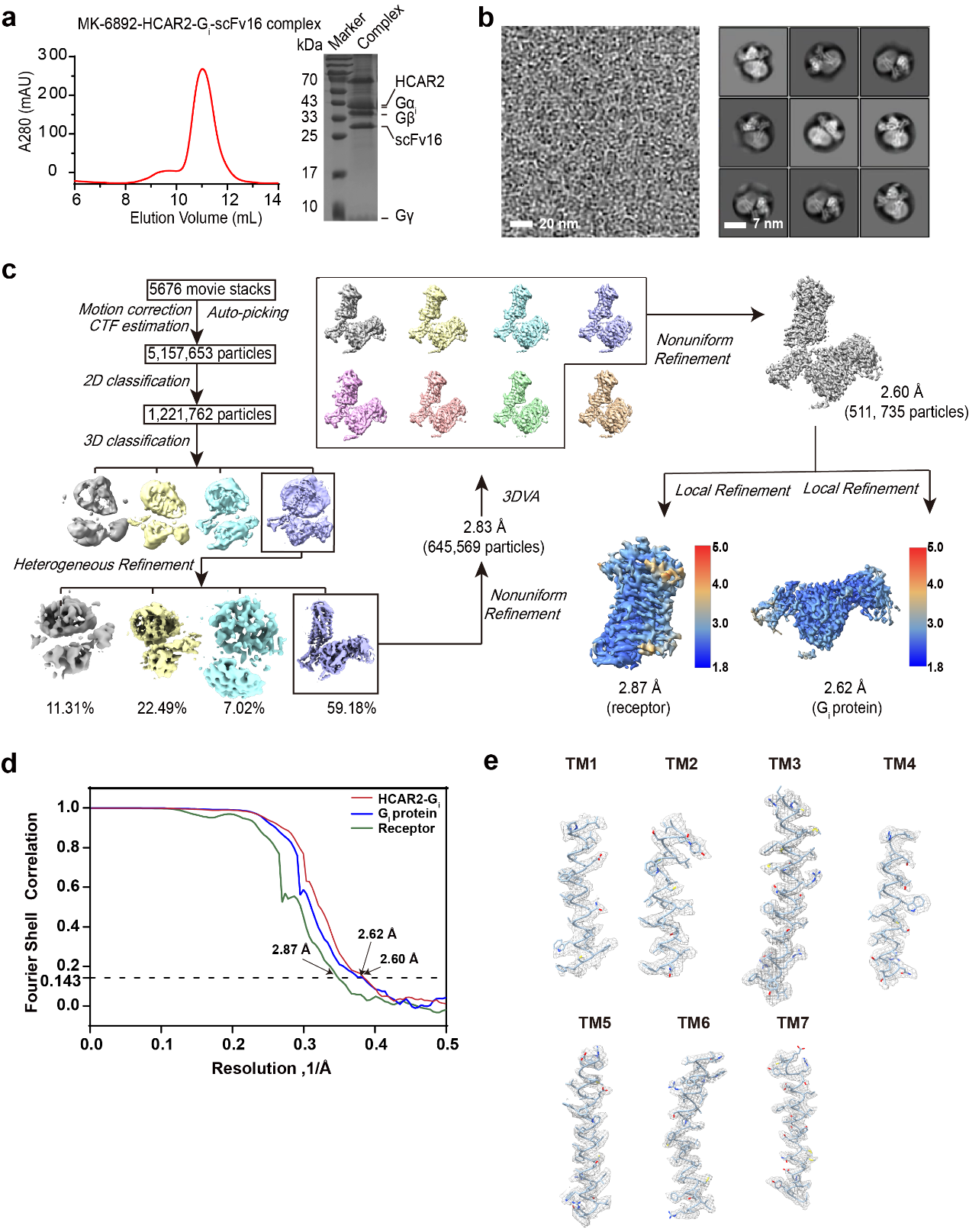


**Supplementary Fig. S1. Data processing of MK-6892-HCAR2-G_i_ complex. a** Size-exclusion chromatography elution profile and SDS-PAGE analysis of MK-6892-HCAR2-G_i_ complex. **b** Representative cryo-EM micrographs (left panel) and 2D classification averages (right panel) of MK-6892-HCAR2-G_i_ complex. **c** Cryo-EM data processing flowchart of MK-6892-HCAR2-G_i_ complex. EM-map with global resolution of 2.60 Å and local resolution range from 1.8 Å to 5.0 Å were acquired from 511,735 particles. **d** Gold-standard Fourier shell correlation (FSC) curve of MK-6892-HCAR2-G_i_ complex, HCAR2 receptor and G_i_ protein calculated by CryoSPARC. **e** Cryo-EM density maps and models of the transmembrane helices (TM) regions with the counter of 0.53.


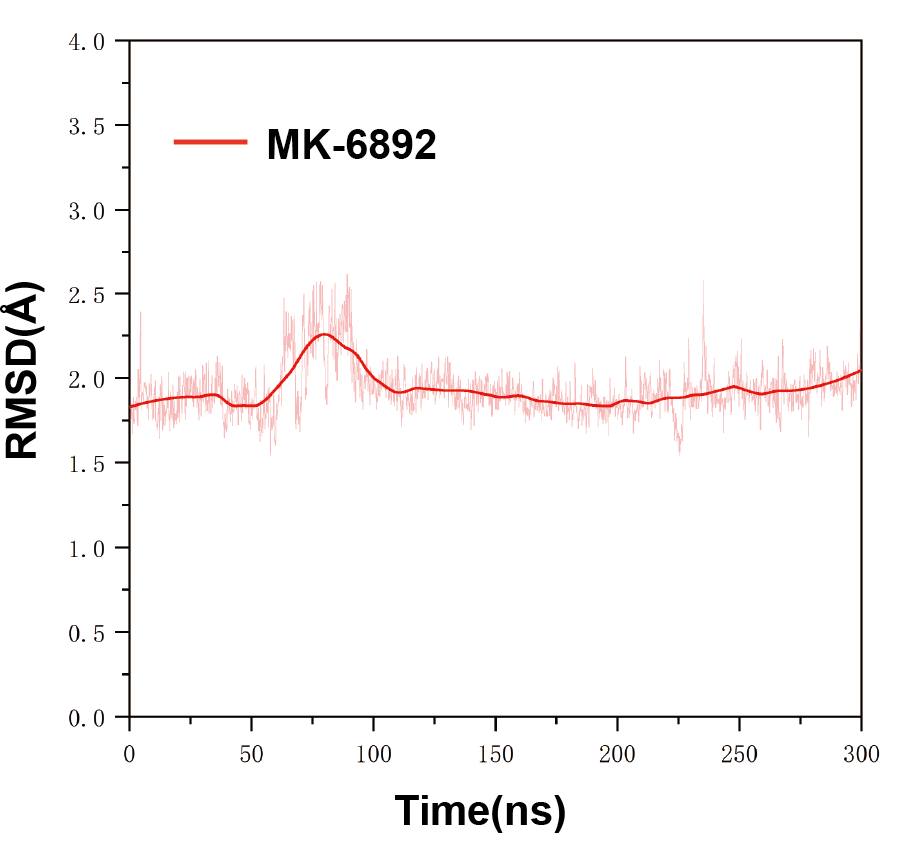


**Supplementary Fig. S2. 300-ns MD simulation course for HCAR2-MK-6892 complex.** Red trace represents for the RMSD values of the ligand MK-6892.


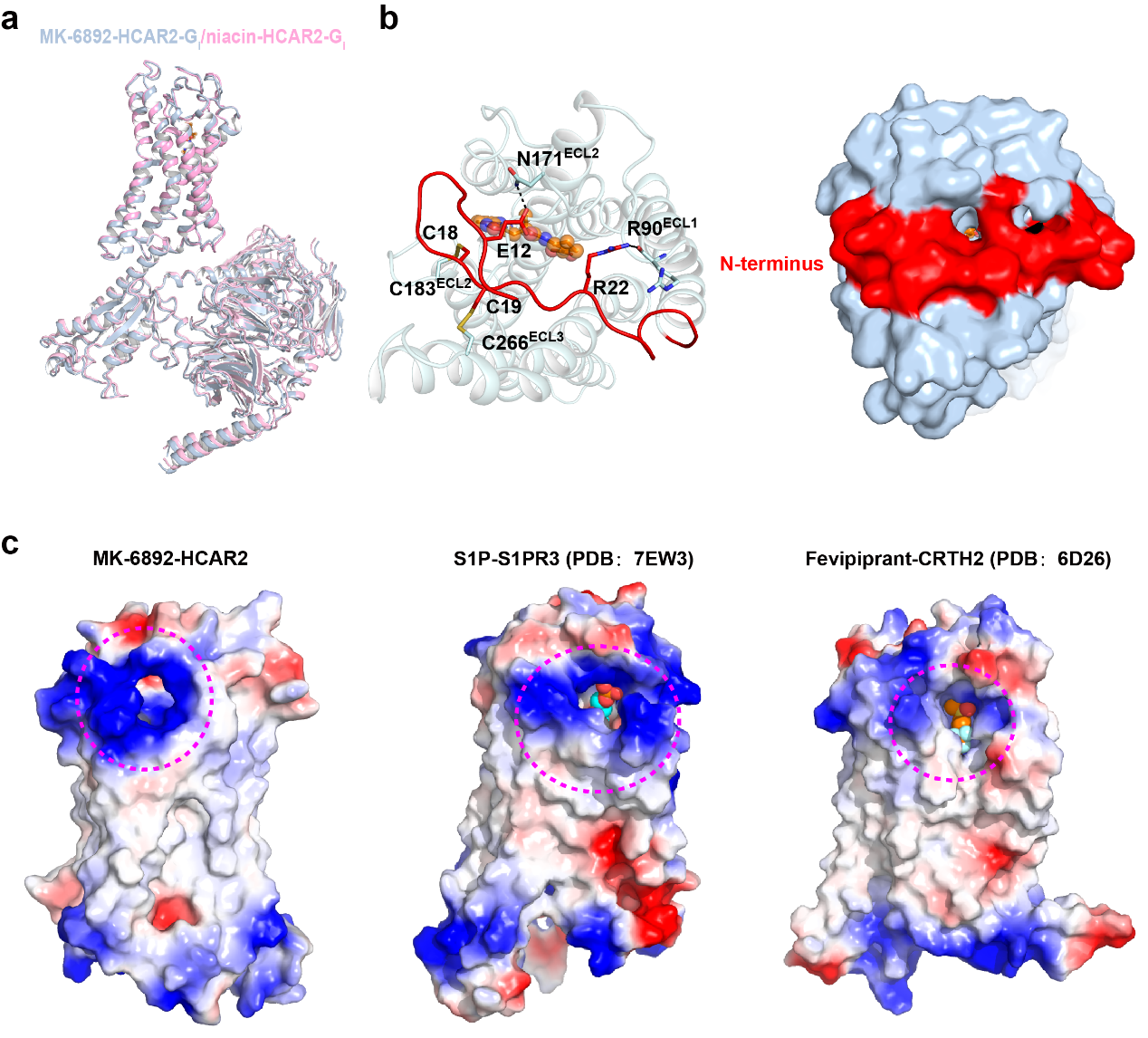


**Supplementary Fig. S3. Structural property of HCAR2. a** Structural superposition of MK-6892-bound (light blue) and niacin-bound (pink) HCAR2-G_i_ complex reveals the two structures exhibit a similar conformation. **b** Top view of the MK-6892-bound HCAR2 complex shows that the N-terminus of HCAR2 forms a cap to close the ligand access channel. **c** Structural comparison of HCAR2, S1PR3 (PDB: 7EW3) and CRTH2 (PDB: 6D26) reveals a similarly positively charged ligand access port. All the structures are shown as electrostatic surface, blue region is positively charged and red region is negatively charged.


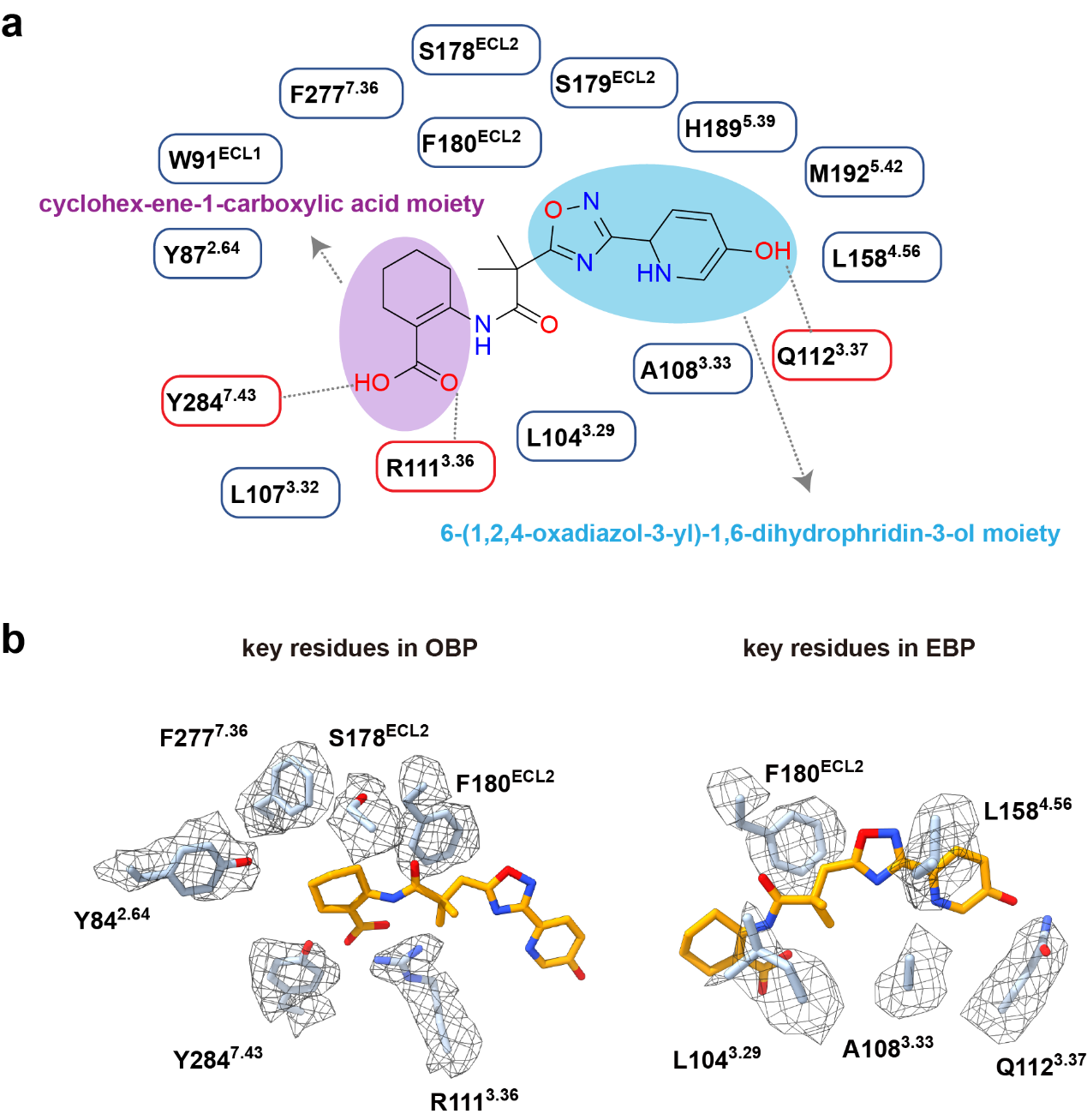


**Supplementary Fig. S4. Binding of MK-6892 to HCAR2. a** 2D diagram of MK-6892 interaction in the ligand binding pocket of HCAR2. The different moieties of MK-6892 binding to the OBP and EBP in HCAR2 are colored with purple and blue respectively. Polar interactions are highlighted as black dashed lines. **b** The EM density of critical residues for MK-6892 binding with the counter level of 0.80.


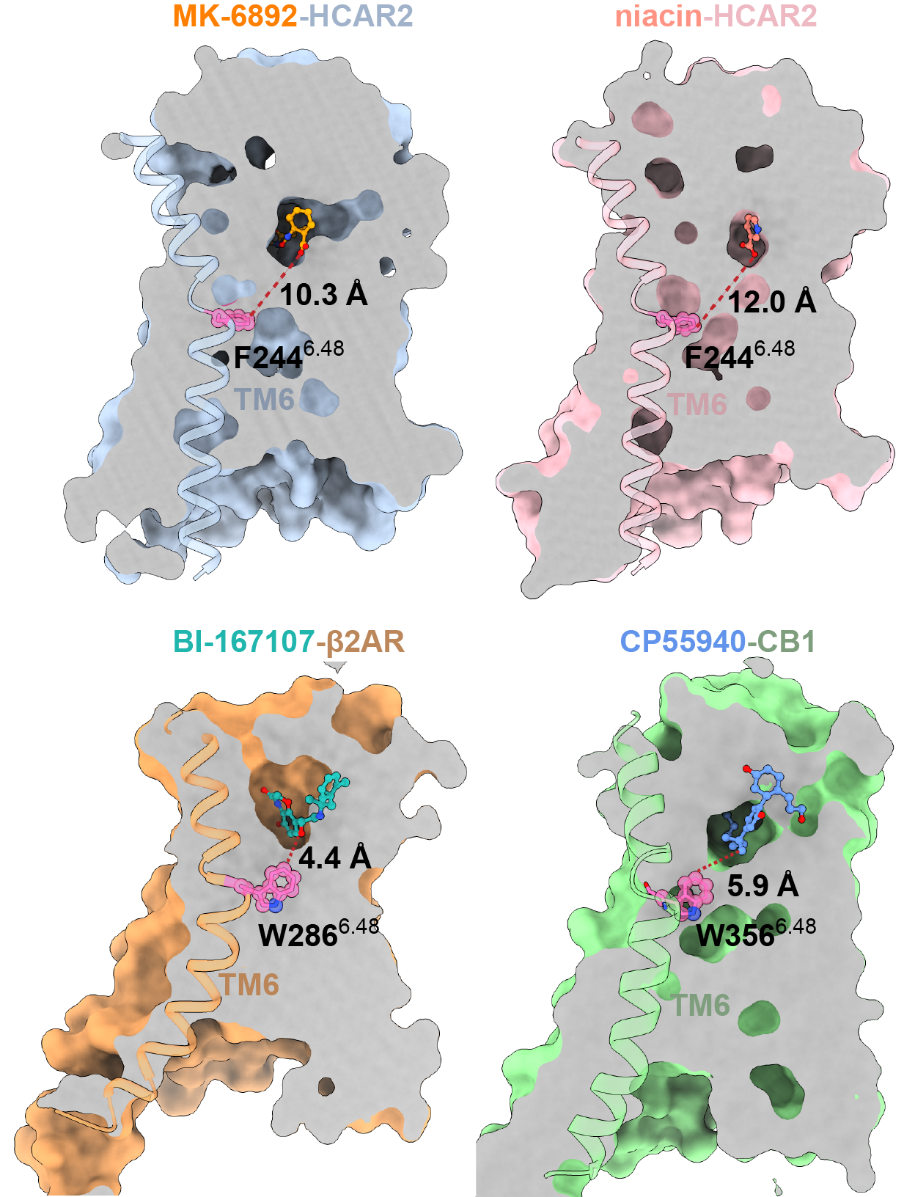


**Supplementary Fig. S5. Activation of HCAR2.** Cut-away view of the ligand binding pocket of MK-6892-bound HCAR2, niacin-bound HCAR2 (PDB: 8JIL), BI-167107-bound β2AR (PDB: 3SN6) and CP55940-bound CB1 (PDB: 7WV9).


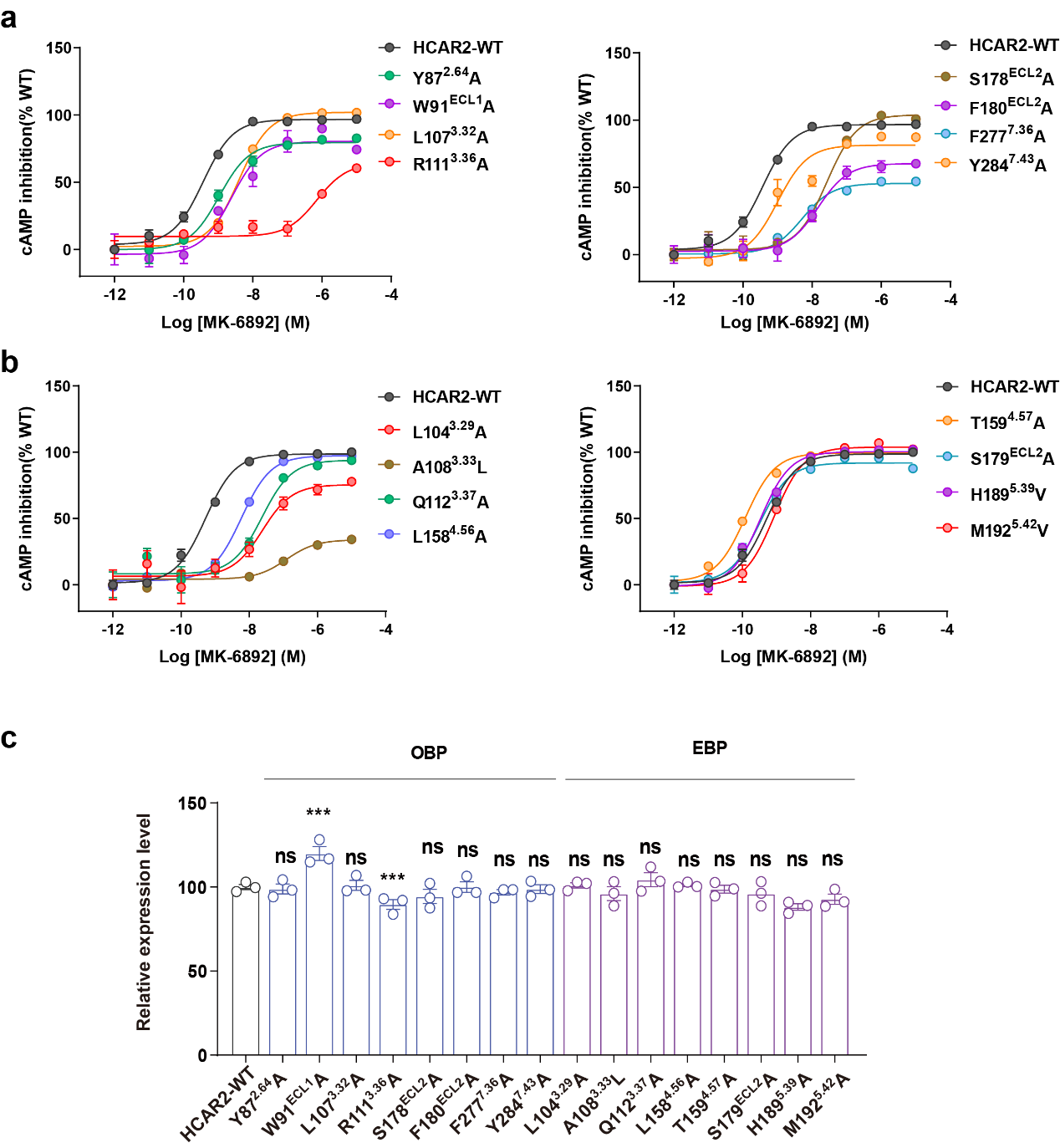


**Supplementary Fig. S6. Effect of mutations in the MK-6892 binding pocket on HCAR2 signaling and surface expression level. a, b** Representative curve for effects of the mutations in HCAR2 on MK-6892 induced G_i_ signaling detected by cAMP inhibition assay. Data are presented as the mean ± SEM of three independent experiments performed in triplicate. **c** The cell surface expression level of the HCAR2 wild-type (WT) and mutants were detected by ELISA assay. Data represent the mean ± SEM from three independent experiments performed in triplicate. ***P<0.001; ns, no significant difference. Data represent the mean ± SEM from n=3 biologically independent experiments performed in triplicate.


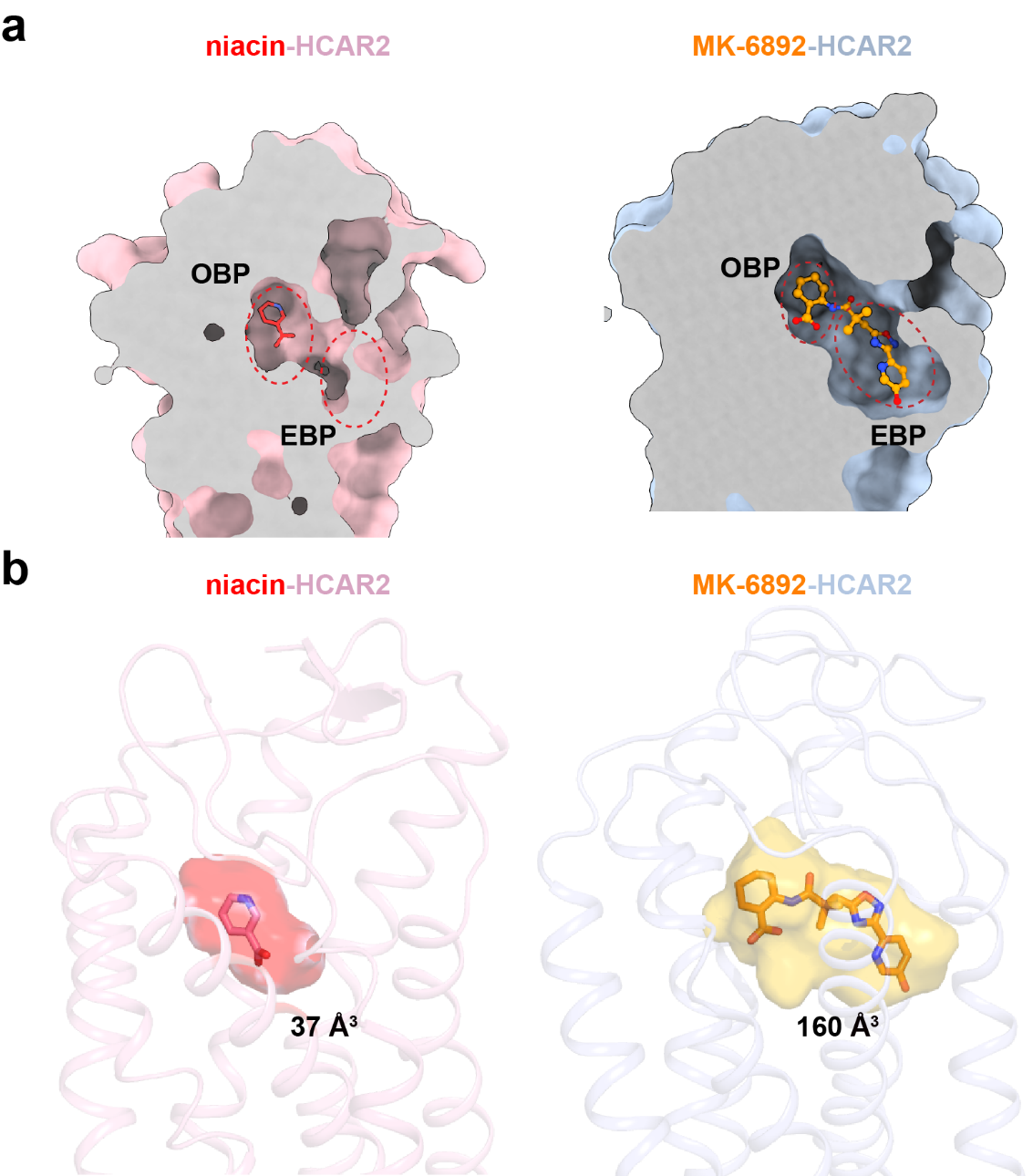


**Supplementary Fig. S7. Comparison of the binding pocket of MK-6892-bound HCAR2 with that of niacin-bound HCAR2. a** Cut-away view of the ligand-binding pocket in the OBP and EBP in the niacin-bound (left panel) and MK-6892-bound (right panel) HCAR2. **b** Comparison of the solvent-accessible volume of the binding pocket for niacin and MK-6892. The solvent-accessible volume was calculated in CASTp 3.0 server.


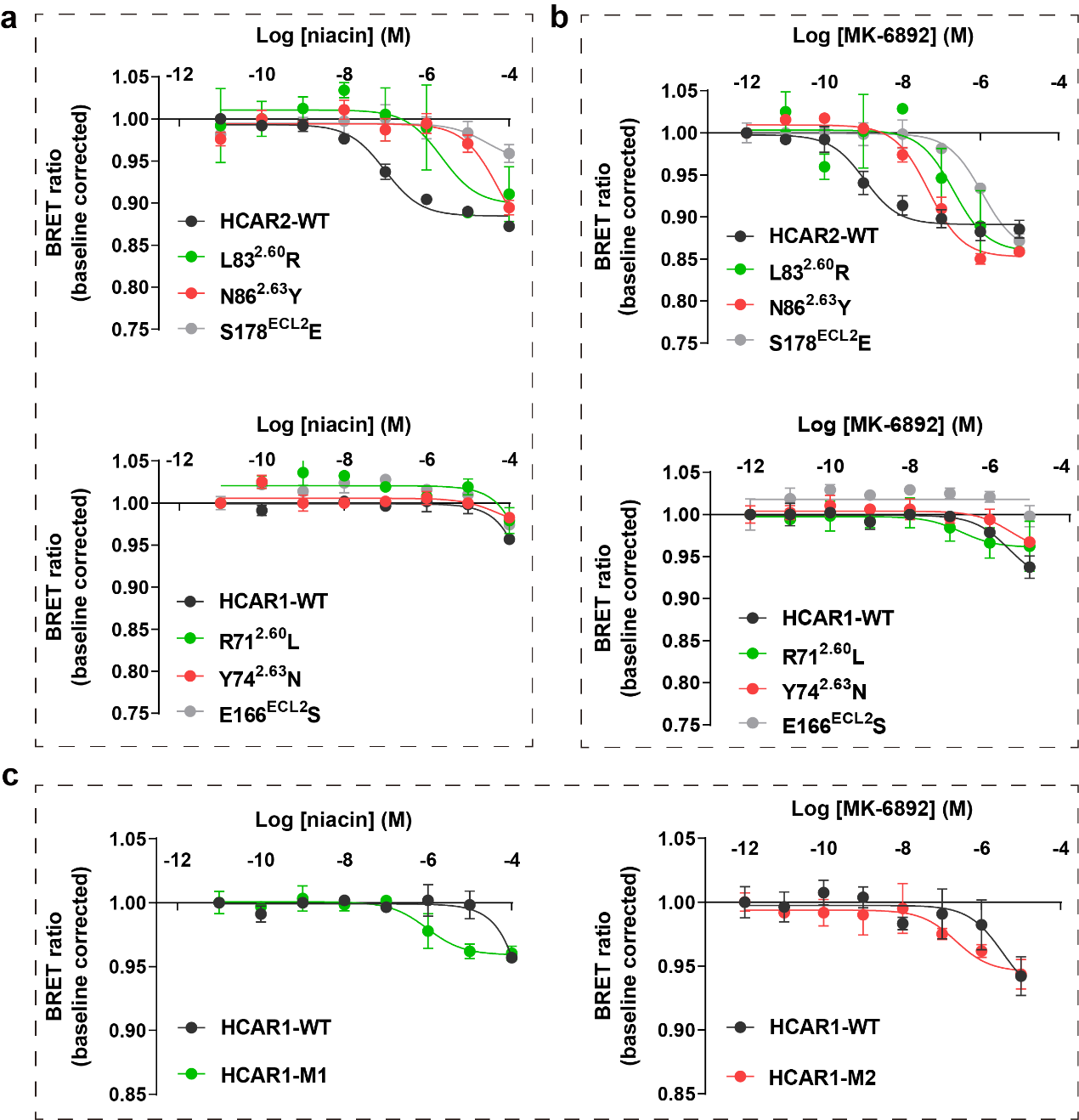


**Supplementary Fig. S8. Selectivity of niacin and MK-6892 on HCAR1 and HCAR2.** **a, b** The Gα_i1_-γ_2_ dissociation BRET assay to examine effects of niacin (**a**) and MK-6892 (**b**) on the swapped residues in HCAR1 and HCAR2 mutants (single point mutation), respectively. Data are presented as the mean ± SEM of three independent experiments performed in triplicate. **c** Effects of multipoint mutation on HCAR1 signaling induced by niacin (left panel) or MK-6892 (right panel). M1 indicates the R^2.60^L/Y^2.63^N/R^ECL1^W/E^45.50^S mutant, M2 indicates the R^2.60^L/Y^2.63^N/R^ECL1^W/E^45.50^S/A^3.37^Q/G^4.57^L mutant. Data are displayed as mean ± SEM from n=3 biologically independent experiments, each performed in triplicate.

**
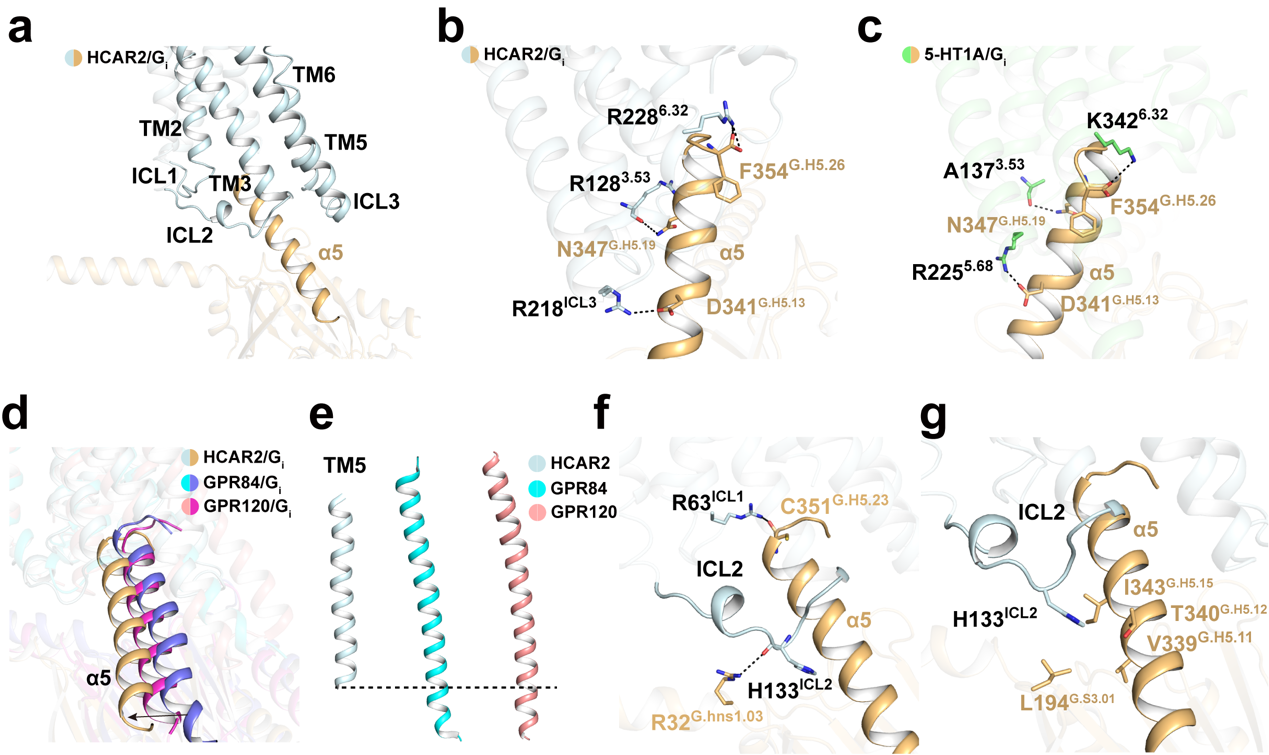
**

**Supplementary Fig. S9. Structure of HCAR2-G_i_ complex. a** HCAR2 interacts with the G_i_ protein mainly by TM2, TM3, TM5, TM6 and intracellular loops (ICL1, ICL2, ICL3). **b, c** The common polar interactions identified in HCAR2-G_i_ complex (**b**) and 5-HT1A-G_i_ complex (**c**, PDB: 7E2X). Polar interactions are highlighted as black dashed lines. **d, e** Structural comparison of HCAR2-, GPR84 (PDB: 8J18)-, GPR120 (PDB: 8G59)-bound G_i_ complex. In HCAR2-bound G_i_ complex, the α5 helix of G_i_ displays a notable displacement (**d**) and HCAR2 shows a short TM5 (**e**). **f** The detailed polar interactions between G_i_ and intracellular loops (ICL1, ICL2) of HCAR2. **g** The residue H133^ICL2^ inserts into a hydrophobic cavity constituted by the residues in G_i_.


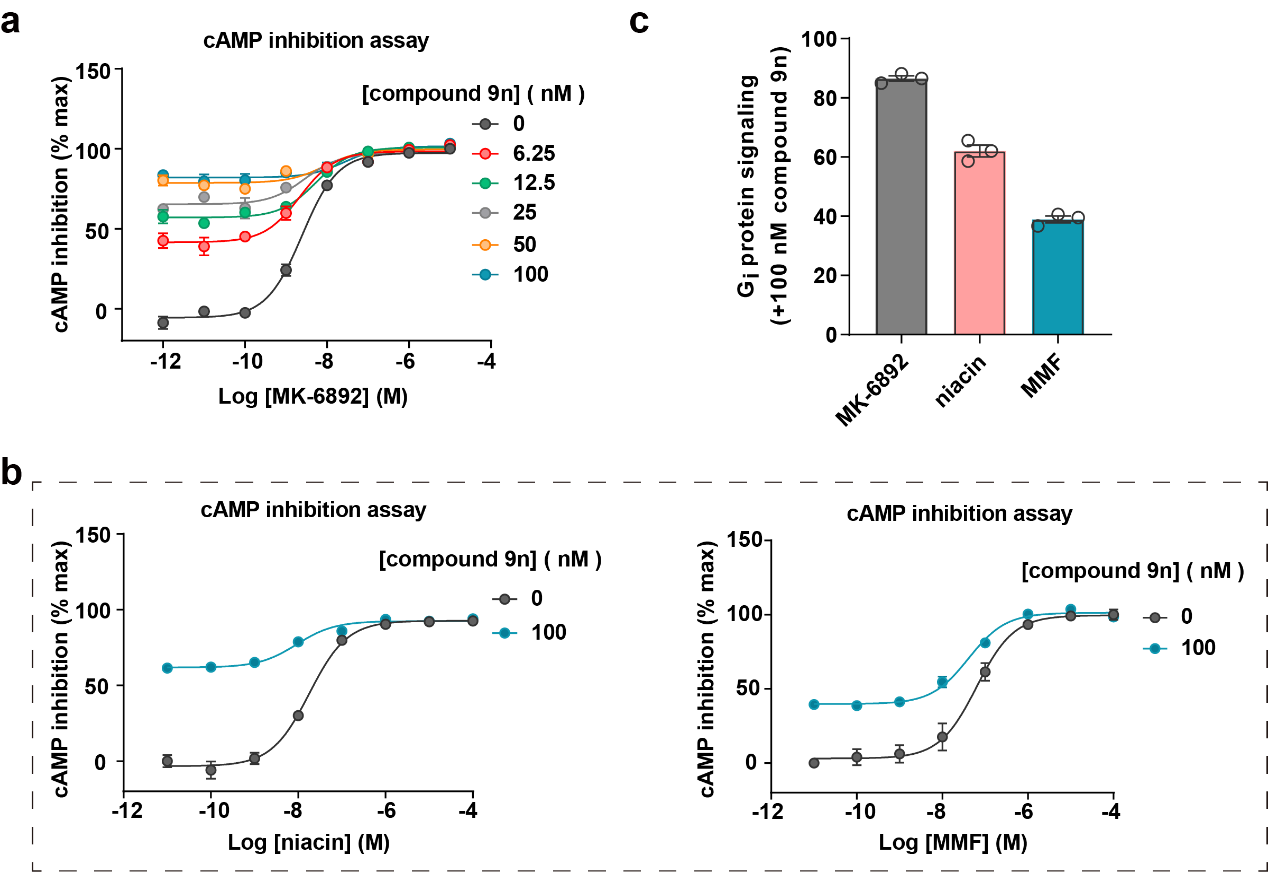


**Supplementary Fig. S10. The allosteric effect of compound 9n on HCAR2 downstream G_i_ protein signaling. a, b** The allosteric effect of compound 9n on HCAR2 downstream signaling induced by MK-6892 (**a**), niacin (**b**, left panel) and MMF (**b**, right panel). The G_i_ signaling was determined by cAMP inhibition assay. Dose dependent curves were shown. Data are presented as the mean ± SEM of three independent experiments, each performed in triplicate. **c** Comparison of the allosteric effect of compound 9n on HCAR2-WT downstream G_i_ protein signaling induced by MK-6892, niacin and MMF. The cAMP inhibition is calculated from data presented in curves of allosteric effect in the presence of 100 nM compound 9n.

**Supplementary Table S1 | Cryo-EM data collection, refinement and validation statistics of MK-6892-HCAR2-G_i_-scFv16 complex.**

| **Parameters** | **MK-6892-HCAR2-G_i_-scFv16** |
| --- | --- |
| **Data collection and processing** |  |
| Magnification | 130,000 |
| Voltage (kV) | 300 |
| Electron exposure (e^–^/Å^2^) | 65 |
| Defocus range (μm) | -1 to -1.8 |
| Pixel size (Å) | 0.92 |
| Symmetry imposed | C1 |
| Initial particle images (no.) | 5,157,653 |
| Final particle images (no.) | 511,735 |
| Map resolution (Å) | 2.60 |
| FSC threshold | 0.143 |
| Map resolution range (Å) | 1.8-5.0 |
| **Refinement** |  |
| Initial model used (PDB code) | niacin-HCAR2-G_i_ complex |
| Model resolution (Å) | 3.3 |
| FSC threshold | 0.5 |
| Map sharpening B factor (Å^2^) | -115.6 |
| Model composition |  |
| Non-hydrogen atoms | 9042 |
| Protein residues | 1144 |
| Ligands | 1 |
| Lipids | 0 |
| B factors (Å) |  |
| Protein | 68.85 |
| Ligands | 66.60 |
| R.m.s. deviations |  |
| Bond lengths (Å) | 0.004 |
| Bond angles (°) | 1.133 |
| Validation |  |
| MolProbity score | 2.13 |
| Clash score | 15.03 |
| Poor rotamers (%) | 0.00 |
| Ramachandran plot |  |
| Favored (%) | 93.01 |
| Allowed (%) | 6.99 |
| Disallowed (%) | 0 |

**Supplementary Table S2 | Summary of MK-6892-mediated cAMP inhibition of HCAR2 and its mutations. Related to the Methods section “cAMP inhibition assay”.**

| **Receptors** | **EC_50_ (nM)** | **Fold** | ***E*max (%WT)** | **n** | **Expression level (%WT)** |
| --- | --- | --- | --- | --- | --- |
| **HCAR2-WT** | 0.37±0.03 | 1.00 | 100 | 6 | 100 |
| **Y87^2.64^A** | 1.08±0.06 | 2.92 | 79.45±2.28 | 3 | 98.85±2.94 |
| **W91^ECL1^A** | 2.41±0.09 | 6.51 | 80.33±3.95 | 3 | 119.9±4.16 |
| **L104^3.29^A** | 24.25±0.12 | 65.54 | 75.47±5.09 | 3 | 101±1.57 |
| **L107^3.32^A** | 4.10±0.02 | 11.08 | 102±1.04 | 3 | 101±2.97 |
| **A108^3.33^L** | 122±0.11 | 329.73 | 33.93±2.39 | 3 | 96.1±4.15 |
| **R111^3.36^A** | 720.2±0.11 | 1946.48 | 64.11±6.01 | 3 | 89.63±2.94 |
| **Q112^3.37^A** | 23.85±0.08 | 64.45 | 93.93±4.29 | 3 | 104.3±4.17 |
| **L158^4.56^A** | 5.60±0.02 | 15.13 | 97.36±1.16 | 6 | 101.1±0.93 |
| **T159^4.57^A** | 0.12±0.02 | 0.32 | 99.43±0.76 | 3 | 98.76±2.31 |
| **S178^ECL2^A** | 26.53±0.06 | 71.70 | 104±3.83 | 3 | 94.41±4.24 |
| **S179^ECL2^A** | 0.32±0.04 | 0.86 | 91.89±1.59 | 3 | 96.1±4.15 |
| **F180^ECL2^A** | 14.95±0.09 | 40.41 | 67.69±3.69 | 3 | 100±3.20 |
| **H189^5.39^V** | 0.34±0.03 | 0.92 | 100.3±1.17 | 3 | 88.12±1.98 |
| **M192^5.42^V** | 0.85±0.04 | 2.30 | 103.8±1.89 | 3 | 92.82±3.03 |
| **F277^7.36^A** | 5.15±0.05 | 13.92 | 52.93±1.23 | 3 | 96.81±1.60 |
| **Y284^7.43^A** | 1.01±0.08 | 2.73 | 81.44±3.30 | 3 | 98.76±2.62 |
